# The burden of illness in Lennox–Gastaut syndrome: a systematic literature review

**DOI:** 10.1186/s13023-023-02626-4

**Published:** 2023-03-01

**Authors:** Adam Strzelczyk, Sameer M. Zuberi, Pasquale Striano, Felix Rosenow, Susanne Schubert-Bast

**Affiliations:** 1 Epilepsy Center Frankfurt Rhine-Main, Center of Neurology and Neurosurgery, University Hospital and Goethe-University Frankfurt, Schleusenweg 2-16 (Haus 95), 60528 Frankfurt am Main, Germany; 2 LOEWE Center for Personalized and Translational Epilepsy Research (CePTER), Goethe-University Frankfurt, Frankfurt am Main, Germany; 3 Paediatric Neurosciences Research Group, Royal Hospital for Children, School of Health and Wellbeing, University of Glasgow, Glasgow, UK; 4 IRCCS ‘G. Gaslini’ Institute, Genova, Italy; 5 Department of Neurosciences, Rehabilitation, Ophthalmology, Genetics, Maternal and Child Health, University of Genoa, Genova, Italy; 6 Department of Neuropediatrics, University Hospital and Goethe-University Frankfurt, Frankfurt am Main, Germany

**Keywords:** Burden of illness, Direct costs, Indirect costs, Caregiver burden, Health-related quality of life, Developmental and epileptic encephalopathy, Epilepsy, Lennox–Gastaut syndrome, Seizures

## Abstract

**Background:**

Lennox–Gastaut syndrome (LGS) is a severe developmental and epileptic encephalopathy characterized by drug-resistant epilepsy with multiple seizure types starting in childhood, a typical slow spike-wave pattern on electroencephalogram, and cognitive dysfunction.

**Methods:**

We performed a systematic literature review according to the PRISMA guidelines to identify, synthesize and appraise the burden of illness in LGS (including “probable” LGS). Studies were identified by searching MEDLINE, Embase and APA PsychInfo, Cochrane’s database of systematic reviews, and Epistemonikos. The outcomes were epidemiology (incidence, prevalence or mortality), direct and indirect costs, healthcare resource utilization, and patient and caregiver health-related quality of life (HRQoL).

**Results:**

The search identified 22 publications evaluating the epidemiology (n = 10), direct costs and resource (n = 10) and/or HRQoL (n = 5). No studies reporting on indirect costs were identified. With no specific ICD code for LGS in many regions, several studies had to rely upon indirect methods to identify their patient populations (e.g., algorithms to search insurance claims databases to identify “probable” LGS). There was heterogeneity between studies in how LGS was defined, the size of the populations, ages of the patients and length of the follow-up period. The prevalence varied from 4.2 to 60.8 per 100,000 people across studies for probable LGS and 2.9–28 per 100,000 for a confirmed/narrow definition of LGS. LGS was associated with high mortality rates compared to the general population and epilepsy population. Healthcare resource utilization and direct costs were substantial across all studies. Mean annual direct costs per person varied from $24,048 to $80,545 across studies, and home-based care and inpatient care were significant cost drivers. Studies showed that the HRQoL of patients and caregivers was adversely affected, although only a few studies were identified. In addition, studies suggested that seizure events were associated with higher costs and worse HRQoL. The risk of bias was low or moderate in most studies.

**Conclusions:**

LGS is associated with a significant burden of illness featuring resistant seizures associated with higher costs and worse HRQoL. More research is needed, especially in evaluating indirect costs and caregiver burden, where there is a notable lack of studies.

**Supplementary Information:**

The online version contains supplementary material available at 10.1186/s13023-023-02626-4.

## Background

**Fig 1:**
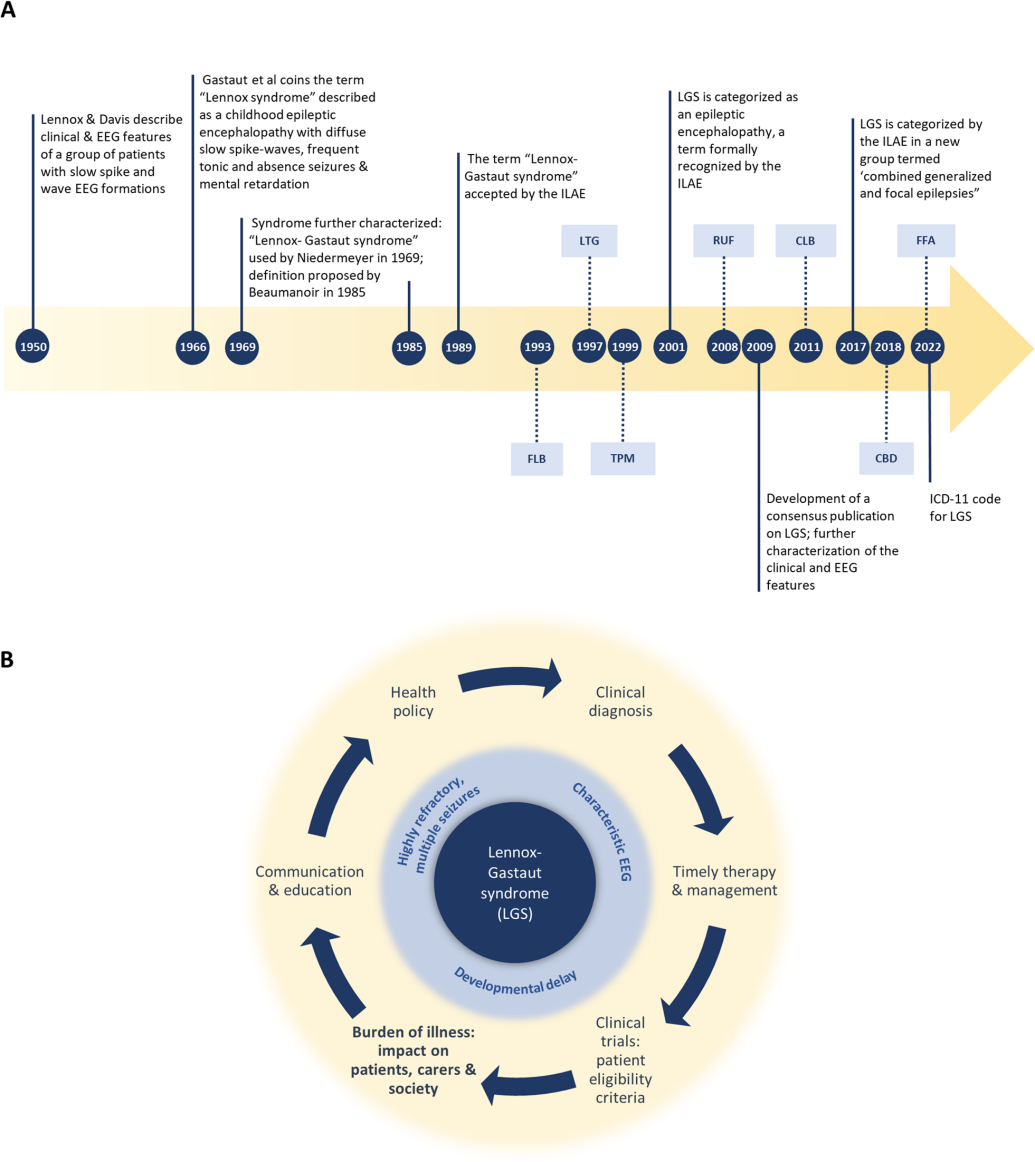
Timeline to show **A** the history of the characterization and treatment of LGS **B** the triad of symptoms characteristic of LGS and the clinical implications of the characterization of the syndrome. From: Lennox et al (1950) [1], Gastraut et al (1966) [2], Niedermeyer (1969) [90], Beaumanoir (1985) [91], ILAE 1989 [92], Engel (2001) [93], Arzimanoglou et al (2009) [3], Scheffer (2017) [94], World Health Organization (ICD-11) (2022) [64]. CLB, clobazam; CBD, cannabidiol; EEG, electroencephalogram; FFA, fenfluramine; FLB, felbamate; ILAE, International League Against Epilepsy; LGS, Lennox–Gastaut syndrome; LTG, lamotrigine; RUF, rufinamide; TPM, topiramate

The recognition of a specific disease entity is vital for every aspect of patient care, from diagnosis, management and treatment to advancements in health outcomes through research and health policy (Fig 1). The definition of Lennox–Gastaut syndrome (LGS) has been evolving since Lennox and Davis (1950) [1] and Gastaut et al (1960) [2] described the electroclinical features of a severe, refractory, childhood epileptic encephalopathy with slow spike and wave electroencephalogram (EEG) formations (Fig 1A). Studies elucidating LGS over the years have established a triad of classical features that can be used in clinical practice and research [3–5] (Fig  1B): 1. Multiple types of drug-resistant seizures, including tonic, atonic, and atypical absences, with onset generally before the age of 8 years (peak age of onset between 3 and 5 years). Tonic seizures and atypical absences are mandatory for diagnosis. 2. Interictal EEG pattern of diffuse, slow spike-wave complexes; and 3. developmental delay. However, importantly, it has also become clear that there are caveats to these features which can make LGS challenging to define and diagnose [5]. For example, none of the 3 features alone is pathognomonic, the main seizure types are not always present, and the features and progression of LGS is variable [6–11]. In addition, LGS is associated with multiple etiologies, as opposed to the known monogenetic causes that can be diagnostic for other epilepsy syndromes such as Dravet syndrome (DS) and tuberous sclerosis complex (TSC). Indeed, LGS can often arise from another severe infantile epilepsy syndrome including infantile spasms syndrome, early infantile developmental and epileptic encephalopathy, epilepsy of infancy with migrating focal seizures and TSC [12].

Despite the challenges of defining LGS, there have been significant clinical improvements over the years [5, 13–16]. There are now seven anti-seizure medications (ASMs) specifically indicated in patients with LGS in the US [15]—clobazam, felbamate, lamotrigine, topiramate, rufinamide, cannabidiol and fenfluramine, with efficacy demonstrated in clinical trials for seizures associated with drops in LGS patients (Fig 1A) [17–23]. Other interventions include the ketogenic diet, vagus nerve stimulation, and corpus callosotomy [5, 13–16].

The term “burden of illness” encompasses outcomes including the epidemiology (prevalence/incidence and mortality), healthcare resource utilization, costs, and patient and caregiver health-related quality of life (HRQoL) [24, 25]. In addition to clinical research investigating therapies, evaluating the burden of illness is key to understanding the challenges and needs of patients and their caregivers, together with understanding the impact and challenges on healthcare systems and society. Systematic literature reviews (SLRs) evaluating the burden of illness in DS and TSC have recently been published [25, 26]. However, there are no SLRs on this topic for LGS.

We conducted an SLR with a narrative synthesis to comprehensively identify, synthesize and appraise the evidence associated with the burden of illness in individuals with LGS, as well as to identify gaps in the evidence. The focus of this SLR was on the overall burden as opposed to the impact of specific interventions.

## Methods

We carried out an SLR following the Preferred Reporting Items for Systematic Reviews and Meta-Analyses (PRISMA) guidelines [27]. The protocol was registered with Prospero (CRD42022333410).

### Literature search

We searched MEDLINE, Embase and APA PsychInfo (all via OVID), Cochrane’s database of systematic reviews (CDSR, Wiley), and Epistemonikos. The search strategy took the following form: ((terms for Lennox–Gastaut syndrome) AND (modified search filters for epidemiology, resource use, and health-related quality of life)) and it was developed by an information specialist and checked by the authors [28–31]. Conference proceedings were searched via Embase and we checked the reference lists of eligible studies and relevant systematic reviews [32]. The search was not limited by language and it was reported in web-only material using a search narrative [33]. The search terms used to identify the relevant literature can be seen in the Additional file 1: (Supplementary information: Search terms). The databases were searched from inception to 10th September 2021.

### Eligibility criteria

A summary of the eligibility criteria for this review according to population, intervention, comparator, outcomes and study design (PICOS) is presented in Table 1. Eligible studies were those that reported original data on the above outcomes without the impact of an intervention. In vitro and in vivo studies, preclinical studies, review articles, SLRs, editorials, and case studies were not eligible, however the reference lists of reviews and SLRs were searched to ensure all relevant studies were captured. In addition, economic evaluations (e.g cost-effectiveness, cost-utility and budget impact models) reporting on specific interventions without any original data on e.g HRQoL (e.g utilities) and healthcare resource utilization were excluded, however the reference lists were searched for publications containing original data. No limits were applied (e.g regarding publication dates or language), except for conference abstracts that were limited to the previous 3 years.

**Table 1:** PICOS criteria for inclusion and exclusion of studies

PICOS	Inclusion	Exclusion
Population	Patients with LGS, including clearly defined “probable” LGS	Other DEEs
Intervention	Studies assessing the overall burden of illness of LGS without the impact of an intervention	Studies assessing the impact of interventions^a^
Comparator	N/A	–
Outcomes	Prevalence/incidence Economic burden Direct and indirect costs Resource use Patient and caregiver HRQoL and broader patient-reported outcomes Mortality	–
Study designs/publication type	Publications reporting original data, including: Healthcare insurance claims data Chart reviews Registry studies Electronic healthcare databases studies Epidemiologic surveys Observational studies Economic evaluations if they contain original data on resource utilisation and HRQoL	*In vitro* and *in vivo* studies Preclinical studies Case reports and case series Clinical studies reporting only efficacy and safety data Economic evaluations reporting on specific interventions^b^ Reviews^c^, SLRs^c^, comments, letters, editorials and press releases
Limits	Searches of conferences in Embase were limited to the previous 3 years (conferences 2018-current)^d^	None

HRQoL, health-related quality of life; LGS, Lennox–Gastaut syndrome; N/A, non-applicable; SLR, systematic literature review

^a^The focus is on the overall burden as opposed to the impact of specific interventions

^b^Economic evaluations reporting on specific interventions without any original data on e.g HRQoL (utilities) and healthcare resource utilisation will be excluded, however, the reference lists will be searched to ensure all relevant studies are captured in this SLR.

^c^Reviews and SLRs will be excluded from final inclusion but the reference lists will be searched to ensure all relevant studies are captured in this SLR.

^d^The rationale for excluding conference abstracts > 3 years is that this should be sufficient time for the data to be published in peer-review journals if they are of sufficient importance and quality. The rationale for including conference abstracts ≤3 years is to include the most up-to-date data.

### Study selection

Publications identified via the bibliographic database searches were managed and screened in Covidence, which is an online tool that helps to facilitate the systematic review process [34]. To ensure the accuracy of the screening process, two independent reviewers assessed relevant articles. Initially, primary screening was performed by reviewing the title and abstract and articles were excluded if they were clearly not relevant according to the PICOS eligibility criteria (e.g if the article was about a different condition and clearly not about patients with LGS etc). For all potentially relevant articles, full articles were obtained, and secondary screening was performed by reviewing the full text against the PICOS criteria. The reasons for exclusion at secondary screening were clearly documented according to the PICOS criteria. Disagreements regarding the inclusion and/or exclusion of studies at both primary and secondary screening were generally resolved by discussion between the two reviewers, or if needed by a third reviewer.

### Data extraction and synthesis

Data were extracted into tables in MS Word by one reviewer, which was checked for accuracy by a second independent reviewer. The prespecified variables were as follows: Study and population characteristics i.e study type, observation period, population definition and number, age (mean, standard deviation [SD], median, range), percentage of male patients, and outcomes reported; epidemiology outcomes i.e incidence, prevalence and mortality as reported by the authors; healthcare resource utilization (mean and SD per person per year [PPPY]) as reported by the authors including inpatient, outpatient, home nursing, equipment, physiotherapy, medications; annual hospitalisation rates; length of hospital stay; anti-seizure medication use; direct costs i.e total direct costs and a breakdown of costs for individual healthcare resources as reported by the authors (mean and SD PPPY); indirect costs (e.g costs from loss of productivity). In addition, quantitative HRQoL measures for patients or caregivers (measure used, patient or caregiver and value) and qualitative HRQoL studies were described descriptively. Data were extracted for patients with LGS, LGS subpopulations (patients with and without seizures and with and without rescue medication) and control populations used by the authors (e.g age matched non-LGS patients).

During the development of the protocol (Prospero: CRD42022333410), it was anticipated that the studies identified in this SLR would be diverse with regard to study design, definition of LGS (especially as there is no specific ICD-9 or 10 code for LGS in some regions and diagnosis can be challenging), size of the populations, the ages of the patients and the length of the follow-up period, and therefore the synthesis of the data was planned to be narrative in nature, without any statistical analyses/comparisons (e.g meta-analyses). Costs were extracted as given in the source. Summaries of the costs across the different studies were provided in the narrative synthesis in US dollars ($) using a simple conversion from Euros (€) to US dollars at an exchange rate of €1.00 Euro =$1.06 (xe.com on 21st June 2022).

### Quality assessment/risk of bias

The quality assessment of the prevalence studies was performed using a tool developed by Hoy et al. [35]. The quality of the cost of illness studies was assessed using a tool from the British Medical Journal Checklist for economic submissions [36] that was adapted by Molinier et al. [37]. The quality assessment of the qualitative and quantitative HRQoL studies was conducted using the Critical Appraisal Skills Programme, CASP; https://casp-uk.net/ that was adapted by Gallop et al. [38].

## Results

### Search results

**Fig 2:**
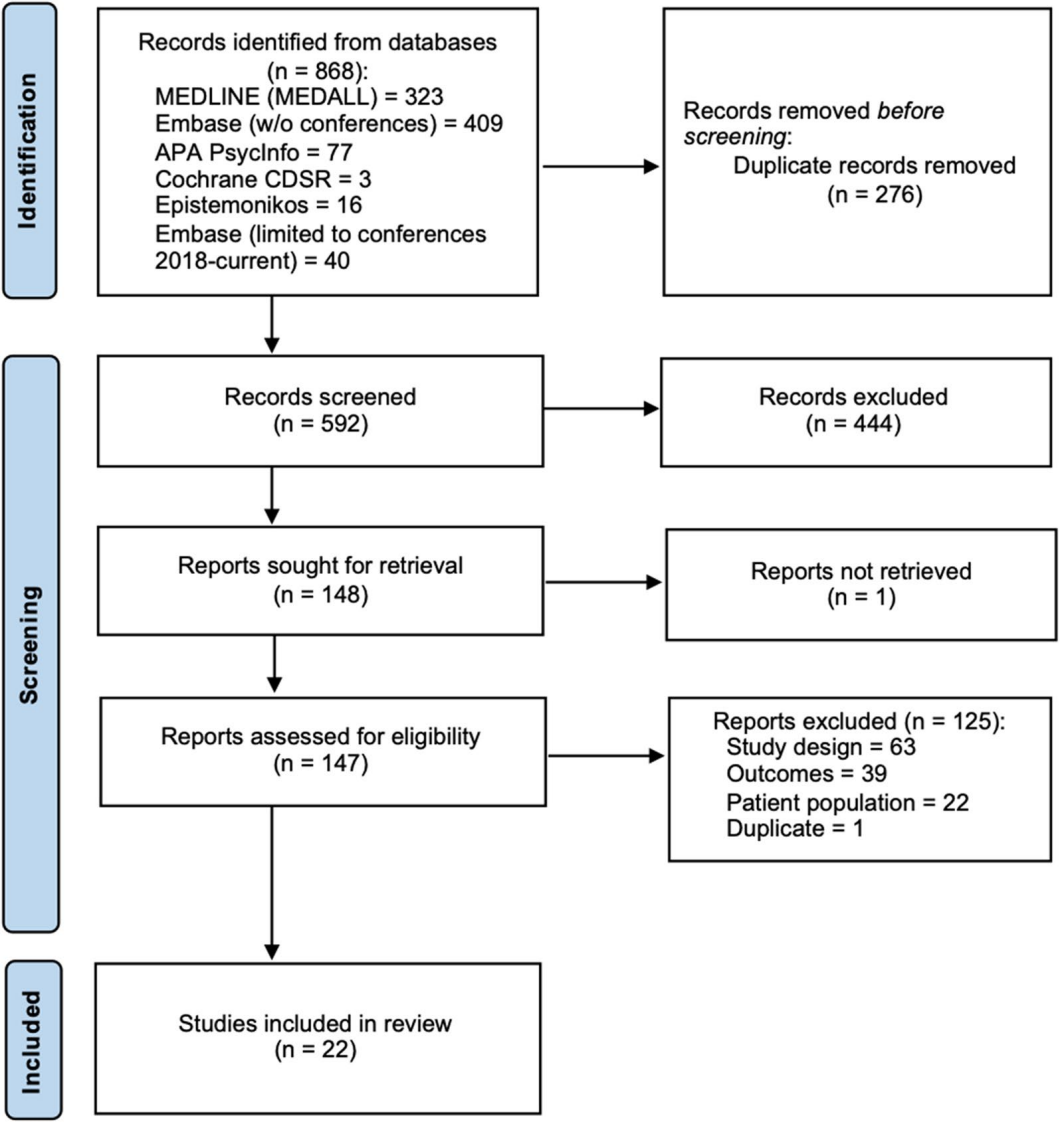
PRISMA flow diagram. Adapted from Page et al (2021) [95]

The electronic database searches initially identified 868 articles, of which 595 were screened after the removal of duplicates. After the primary screening, a total of 149 articles were retrieved for secondary screening (full-text assessment), and of these, 22 met the eligibility criteria (Fig 2). The study characteristics are summarized in Table 2, epidemiology data are summarized in Table 3 and the costs and resource data are presented in Additional file 2: Table S1. The results of the quality assessments/risk of bias are presented in Additional file 2: Tables S2–5.

**Table 2:** Study characteristics

Study, year (Publication type) Country Database	Study type and source of data	Time period	Population: Number	Definition of LGS	Characteristics: Age, mean (SD; median [range]), years; Male, %	Outcomes
*Burden-of-illness/costs and resource*						
Piña-Garza [46] (Full) USA MEDLINE and Embase	Retrospective insurance claims analysis: Medicaid multi-state database of six states	Observation period: mean (SD): 11.1 (4.5) years (1997/98–2012/13)	*Probable LGS*: N=14,712	≥2 medical claims ≥30 days apart for specified epilepsy or unspecified epilepsy (ICD-9-CM codes) Machine-learning technique based on a number of clinical variables (including use of rufinamide during the testing & training period)	16.3 (15.7); 52.5% male	Epidemiology Resource Direct costs
Strzelczyk 2021 [47](Full) Germany MEDLINE, Embase and APA PsycInfo	Retrospective insurance claims analysis: Vilua Healthcare research database, entries for >4 million people i.e ~5% of the German population	10 years (2007–2016)	*Broad LGS:* N=545 *Narrow LGS:* N=102	*Broad definition*: Algorithm considering: ≥1 ICD-10 diagnosis of G40 (epilepsy)/G41 (status epilepticus) and ≥1 claim of either rufinamide or felbamate (during the year of identification), OR ≥2 different ASMs in combination with ≥1 developmental delay diagnosis and no diagnosis code for competing etiologies *Narrow definition*: ≥1 documented G40/G41 claim before their 6th birthday	*Broad LGS:* 31.4 (2–89); 53% male *Narrow LGS:* 7.4 (2–14); 52% male	Epidemiology Mortality Resource Direct costs
Chin 2021 [40] (Full) UK MEDLINE and Embase	Retrospective analysis using electronic medical records from healthcare databases: Primary care data (CPRD) linked to secondary care data (HES), and general population mortality data (ONS)	~31 years (1987–2018) Follow-up: mean (SD; median [range]): 11.7 (8.2; 9.6 [0.2; 32.2]) years	*Confirmed LGS*: N=110 *Probable LGS*: N=146 *Full cohort:* N=256	*Confirmed LGS*: CPRD read code for LGS *Probable LGS*: ICD-10 code/ CPRD read code for epilepsy Rufinamide within a year of diagnosis	*Confirmed LGS*: 7.0 (10.0; 3.5 [0–61]); 66% male *Probable LGS:* 8.9 (11.0; 4 [0–54]); 56% male *Full cohort:* 8.1 (10.6; 4 [0–61]); 60% male	Epidemiology Mortality Resource
Reaven 2018 [54] (Full) (LGS vs controls) USA MEDLINE, Embase and APA PsycInfo	Retrospective insurance claims analysis: Truven Health Analytics MarketScan® Research Databases, entries for ~60 million people with commercial or Medicaid insurance coverage	2010–2015	*Probable LGS*: *Commercial:* N=2270 *Medicaid:* N= 3749	A diagnosis code for refractory epilepsy and A diagnosis code for developmental delay/intellectual disability and A prescription for at least one selected ASM. Excluded patients with diagnosis codes for conditions suggestive of other etiologies Modified version of the algorithm developed by Piña-Garza et al [46] (above)	*Commercial:* 13 (9.8 [0–62]); 53.0% male *Medicaid:* 13 (10.5 [0–60]); 52.9% male	Resource Direct costs
Reaven 2019 [53] (Full) (LGS vs other DEEs; seizure events vs no events) USA MEDLINE, Embase and APA PsycInfo			*Probable LGS*: *Commercial:* N=2269 *Medicaid:* N= 3730		*Commercial:* 13 (9.8 [0–62]); 53.0% male *Medicaid:* 13 (10.5 [0–60]); 52.8% male	
Stockl 2019 [56] (Abstract) (Hospitalizations) USA Embase			*Probable LGS*: *Commercial:* N=2520 *Medicaid:* N=4613	As above, but also patients with an acute inpatient hospitalization (≥1 day LOS)	NR	Resource
Stockl 2019 [55] (Abstract) (Commercial; Costs & Resource) USA Embase	Retrospective insurance claims analysis: IBM® MarketScan® Commercial & Medicaid databases	2015–2016	*Probable LGS*: *Commercial:* N=1296	≥1 ASM claim and medical claims with ≥1 diagnosis code for LGS or refractory epilepsy or ≥1 claim for clobazam or rufinamide. The LGS cohort required a diagnosis code for LGS or all of the following: oooooo≥1 diagnosis code for refractory epilepsy oooooo≥1 diagnosis code for intellectual disability/developmental delay, ooooooNo diagnosis codes that preclude LGS	*Commercial:* 16.5; NR	Epidemiology Resource (ASM use only) Direct costs
Hollenack 2019 [52] (Abstract) (Medicaid; Costs & Resource USA Embase		2014–2015	*Probable LGS*: *Medicaid:* N= 5186		*Medicaid:* 18.3; 58.2% male	
Hollenack 2019 [41] (Abstract) (prevalence) USA Embase		*Commercial:* 2016– 2017 *Medicaid:* 2016	*Probable LGS: Commercial:* N=2273 *Medicaid:* N=4786		NR	
François 2017 [51] (Full) USA MEDLINE	Retrospective insurance claims analysis: Truven Health MarketScan® claims databases, entries for >40 million people with commercial or Medicaid insurance coverage	2010–2014	LGS pre-clobazam initiation *Commercial/medicare:* N=1384 *Medicaid:* N=1365	≥2 medical claims with a diagnosis of generalized convulsive or non-convulsive epilepsy ≥30 days apart, or ≥1 medical claim with a diagnosis of generalized convulsive epilepsy and ≥1 medical claim with a diagnosis of non-convulsive epilepsy (≥30 days apart (ICD-9-CM codes) ≥1 of the epilepsy diagnosis codes in the primary position ≥1 medical claim with a diagnosis for developmental disorder or cognitive impairment (ICD-9-CM codes)	*Commercial/medicare:* 31.0 (23.2); 49.3% male *Medicaid:* 24.1 (17.9); 51.8% male	Resource Direct costs
Umeno 2019 [57] (Full) Japan Embase	Retrospective study on the use of the welfare system in children with DEEs in Japan	2015–2016	LGS: N=30	Children aged 0 to 16 years diagnosed at the Department of Child Neurology, Okayama University Hospital	NR	Resource (welfare system)
*Epidemiology alone*						
Trevathan 1997 [44] (Full) MADDS study USA MEDLINE and Embase	Population-based, cross-sectional study: (MADDS): a multiple-source, EEG laboratory-based, case ascertainment system across Atlanta	1975–1977	Children with LGS: N=23	Epilepsy Two or more seizure types that included tonic, atonic, atypical absence, and/or myoclonic seizures with multiple falls Interictal EEG that demonstrated slow spike-wave discharges	NR; 73.9% male	Prevalence
Autry 2010 [48] (Full) MADDS study USA		1975–2001	LGS: N=34		NR; NR	Mortality
Sidenvall 1996 [43] (Full) Northern Sweden MEDLINE and Embase	Observational study: Active epilepsy assessed in children aged 0–16 years in an area of northern Sweden with ~ 250,000 inhabitants (~50,000 children)	1985–1986	LGS: N=9	Diagnosed using standard ILAE criteria	NR; NR	Prevalence
Rantala 1999 [42] (Full) Finland MEDLINE and Embase	Retrospective study: Children seen at the Department of Pediatrics University of Oulu	1976–1993	LGS: N=25	At least two types of the most common seizure types (tonic-axial, atonic, and absence seizures) Slow spike waves	13.0 [6.5–17.9]; 56% male	Prevalence and incidence
Heiskala 1997 [45] (Full) Finland MEDLINE and Embase	Retrospective study: Health records from the Helsinki metropolitan area & the province of Uusimaa	1975–1985	LGS: N=75	A minimum of two types of typical though nonspecific epileptic seizures (e.g. astatic seizures, tonic seizures during sleep, and atypical absence seizures) Mental deficiency Bursts of diffuse slow spike-waves in the EEG	NR; NR	Incidence
Beilmann 1999 [39] (Full) Estonia MEDLINE	Observational study: Patients with epilepsy seen at the Tartu University Hospital, sourced through multiple methods	1995 –1997	NR	Diagnosed using standard ILAE criteria	NR; NR	Prevalence
**HRQoL**						
Auvin 2021 [61] (Full) UK and France	Observational study: On-line surveys	2018–2019	Patients and/or caregivers of individuals with DEEs estimated a patient’s health state from vignettes of hypothetical patients	NA	NA; NA	Patient HRQoL: quantitative (VAS)
Radu 2019 [62] (Abstract) NR Embase	Observational study: On-line surveys	2018		NA	NA; NA	Patient HRQoL: quantitative (VAS)
Gallop 2010 [58] (Full) US, UK & Italy MEDLINE, Embase and APA PsycInfo	Observational study: Open-ended interviews and HRQoL instruments	NR	Parents of patients with LGS recruited through support groups and websites (UK and US) and through clinicians (Italy): N=40	NR	12 (4–43); 63% male	Caregiver HRQoL: Quantative (SF-36v2, HADS) and qualitative Patient HRQoL: Qualitative
Murray 1993 [60] (Full) Australia MEDLINE, Embase and APA PsycInfo	Observational study: Questionnaires & in-depth interviews	NR	Parents of patients with LGS who attended a self-help group: N=41	NR	13 (3–34); NR	Caregiver HRQoL: Qualitative
Gibson 2014 [59] (Full) USA MEDLINE, Embase and APA PsycInfo	Observational study: General experience and parent survey	NR	Parents of patients with LGS who had contacted a support hotline: N=96 surveys returned	NR	[3.5–36 years]; NR	Caregiver HRQoL: Qualitative

*ASM* anti-seizure medication; *CPRD* Clinical Practice Research Datalink; DEE, developmental and epileptic encephalopathy; DS, Dravet syndrome; HADS, Hospital Anxiety and Depression Scale; HES, Hospital Episode Statistics; HRQoL, health-related quality of life; ILAE, International League Against Epilepsy; LGS, Lennox–Gastaut syndrome; LOS, length of stay; NA, non-applicable; MADDS, Metropolitan Atlanta Developmental Disabilities Study; NR, not reported; ONS, Office for National Statistics; SD, standard deviation; VAS, visual analogue scale

**Table 3:** Epidemiology: Incidence/prevalence and mortality in patients with LGS

Study	Country	Study type	Year	Population (n)	Incidence/prevalence		Mortality
					Parameter	Value	
Piña-Garza 2017 [46] (Full)	USA	Retrospective claims analysis	2012/13	Probable LGS patients N=14,712	Proportion of epilepsy patients with probable LGS (10-year age groups)	8.4% at age 10 decreasing to 2% in the 60-year-old cohort	NR
Strzelczyk 2021 [47] (Full)	Germany	Retrospective claims analysis	2016	*Broad LGS* N=545 *Narrow LGS* N=102	Prevalence (standardized to German GKV population)	*Broad LGS:* 39.2 per 100,000 people *Narrow LGS:* 6.5 per 100,000 people	*Broad LGS: 10.01% [157 events]* *Narrow LGS:* 2.88% [six events] (p < 0.001 vs control) *Control:* 0.01% [one event]
Chin 2021 [40] (Full)	UK	Retrospective analysis of electronic medical records	2017	*Confirmed LGS*: N=74 for prevalence *Probable LGS*: N=106 for prevalence *Full cohort:* N=180 for prevalence; N=122 for mortality	Prevalence (standardized to UK CPRD population)	*Confirmed LGS*: 2.89 per 100,000 people* *Probable LGS*: 4.20 per 100,000 people* *Full cohort:* 5.78 per 100,000 people*	Crude mortality rate per 1000 person-years: *Confirmed LGS*: 6.12 [11 events] *Probable LGS*: 4.17 [7 events] *Full cohort:* 18 events
Hollenack 2019 [41] (Abstract)	USA	Retrospective claims analysis	2016/ 2017	*Probable LGS: Commercial:* N=2273 *Medicaid: N=*4786	Prevalence	*Commercial:* 13.0 per 100,000 people* *Medicaid:* 60.8 per 100,000 people*	NR
Trevathan 1997 [44] MADDS study (Full)	USA	Population-based, cross-sectional study	1975–1977	LGS: N=23	Prevalence of LGS at age 10 years Proportion of epilepsy patients with LGS	26 per 100,000 people* 4% of epilepsy patients	NR
Autry 2010 [48] MADDS study (Full)			Original cohort: 1975–1977 Follow-up: 1975 to 2001	LGS: N=34	NR	NR	Mortality ratio (95% CI): 13.92 (7.19-24.31) for LGS vs 3.11 (2.39-3.98) for all epilepsy
Sidenvall 1996 [43] (Full)	Northern Sweden	Observational	1985	LGS: N=9	Prevalence N (%) of epilepsy patients with LGS	20 per 100,000 people* n=9 (5.8%) of all epilepsy cases	NR
Rantala 1999 [42] (Full)	Finland	Retrospective	1976–1993	LGS: N=25	Prevalence	28 per 100,000 people (95% CI: 18–41)*	NR
					Annual incidence	1.93 per 100,000 children <15 years (95% CI: 1.25-2.85/100,000)	
Heiskala 1997 [45] (Full)	Finland	Retrospective	1975–1985	LGS (broad definition): N=75 LGS children 0–14 years: N=NR	Annual incidence	Broad definition: 2.1 per 100,000 people Children 0–14 years: 1.9 per 100,000	NR
Beilmann 1999 [39] (Full)	Estonia	Observational	1997	LGS: N=NR	Prevalence	10 per 100,000 people*	NR

*CI* confidence interval; *CPRD* UK Clinical Practice Research Datalink; *GKV* German population covered by statutory health insurance (Gesetzliche Krankenversicherung; GKV); *NR* not reported

*Converted to per 100,000 people

### Epidemiology: incidence/prevalence and mortality

Overall, 9 studies (10 publications) reported on the epidemiology of LGS (Table 3). Prevalence, reported in 7 studies, varied from 4.2–60.8 per 100,000 people across studies for probable LGS and 2.9–28 per 100,000 people for a confirmed/narrow definition of LGS [15, 39–44]. Incidence, reported in 2 studies, was estimated at approximately 1.9 per 100,000 people in both studies [42, 45]. In addition, 3 studies reported on the proportion of LGS among epilepsy patients, varying from 4 to 8.4% during childhood [43, 44, 46] [42, 45]. The risk of bias in the studies reporting on prevalence/incidence was low in 3 studies [40, 41, 47] and moderate in 5 studies [39, 42–45] (Additional file 2: Table S2).

Trevathan et al (1997) reported the lifetime prevalence of LGS at the age 10 years to be 26 per 100,000 people (0.26 per 1000 people) [44]. The study used data from the Metropolitan Atlanta Developmental Disabilities Study (MADDS) that encompassed children aged 10 years who were born from January 1, 1975, through December 31, 1977; children with LGS were identified via multiple sources including public schools, special education programs, hospitals, selected physicians’ offices, and EEG laboratories [44]. More recent studies (2019–2021) have used databases covering large populations including claims databases in the USA [41] and Germany [47] and the Clinical Practice Research Datalink (CPRD) database of health records in the UK [40]. These studies have estimated the prevalence of probable LGS to be 13.0–60.8 per 100,000 people in the USA [41], 39.2 per 100,000 people in Germany [47], and 4.2 per 100,000 people in the UK [40]. Using a more stringent definition of LGS resulted in prevalence rates of 6.5 per 100,000 people in Germany [47] and 2.89 per 100,000 people in the UK [40]. Other older, smaller population-based studies have reported the prevalence of LGS to be 10 per 100,000 people in Estonia [39], 2.1–28 per 100,000 people in Finland [42, 45], and 20 per 100,000 people in Sweden [43]. While LGS is a rare syndrome, Piña-Garza et al. [46] reported that LGS accounts for 8.4% of epilepsy cases at age 10 decreasing to 2% in the 60-year-old cohort in the USA. In addition, Trevathan et al (1997) (MADDS study) [44] reported that it accounted for 4% of childhood epilepsy cases, while the study in Sweden [43] found that among the childhood epilepsy syndromes, 5.8% of patients had LGS (Table 2).

Mortality was reported in 3 studies, although with different methods (mortality ratio, mortality rate as a percentage of patients who died divided by the total number of patients in the study, and mortality rate per 1000 person-years) [40, 47, 48]. Overall, the studies showed that mortality was substantially higher in LGS patients than in children with epilepsy and controls (Table 3) [40, 47, 48]. For example, using the MADDS data source, Autry et al. [48] reported a mortality ratio (ratio of observed-to-expected deaths) of 13.92 (95% confidence interval [CI] 7.19–24.31) for children with LGS versus 3.11 (95% CI 2.39–3.98) for all children with epilepsy over a 26-year follow-up period. The retrospective claims analysis from Germany reported a mortality rate (the percentage of patients who died divided by the total number of patients in the study) over the 10-year study period of 10.01% for patients according to the broad definition of LGS and 2.88% for the narrow definition versus 0.01% for the age- and sex-matched control population (p < 0.001) [47]. Using data from the CPRD (electronic health records) linked to mortality data from the Office of National Statistics (ONS) in the UK, Chin et al. [40] estimated the mortality rates to be 6.12 deaths per 1000 person-years for confirmed LGS and 4.17 deaths per 1000 person-years for probable LGS over a follow-up period of 1796 and 1679 patient-years, substantially higher than the mortality rates for the general population and the epilepsy population (0.6 per 1000 person-years in England in 2018 [49], and 0.9 per 1000 person-years in the UK [50], respectively).

### Healthcare resource utilization and costs

Overall, 9 studies (9 publications), all published from 2017 to 2021, were identified that reported on resource utilization and/or direct costs; 7 reported on both resource utilization and direct costs [46, 47, 51–55] (6 from the USA and 1 from Germany), and 2 reported on resource utilization only (1 from the USA using insurance claims databases and 1 from the UK using electronic health records) [40, 56] (Table 2 and Additional file 2: Table S1). By country, most of the studies were from the USA (7 out of 9 studies). All of the costs studies were retrospective analyses of insurance claims databases and the mean annual direct costs were €22,787 in Germany (n = 1 study), and varied from $28,461 to $80,545 in the USA across studies (n = 6 studies). The risk of bias in the studies reporting on costs and/or resources was assessed to be low in 6 studies [40, 46, 47, 51, 53, 54] and moderate in 3 studies, which were conference abstracts that provided limited information [52, 55, 56] (Additional file 2: Table S3). No study reporting the indirect costs of patients with LGS or their caregivers was identified, although one paper reported that a large proportion of caregivers of patients with LGS struggled to access the available medical expense subsidies in Japan [57] (Table 2).

**Fig 3:**
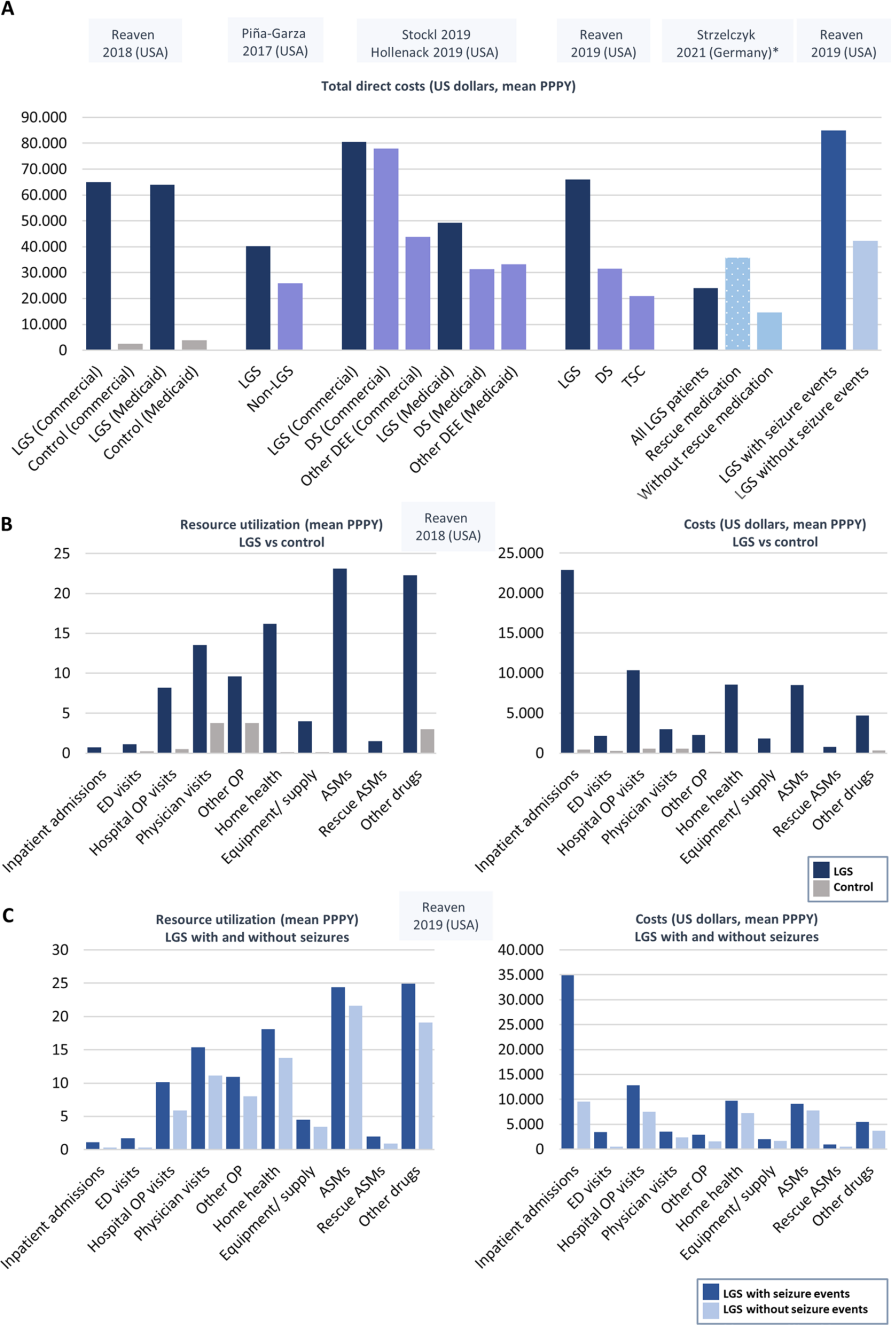
**A** Total direct costs in LGS patients (blue) versus control patients (grey), patients with other epilepsies (lilac), and in LGS patients with seizure events vs without seizure events and LGS patients in years where patients were prescribed with rescue medication vs years where patients were not prescribed rescue medication (blue) **B** resource utilization and costs in LGS patients vs controls; **C** resource utilization and costs in LGS patients with seizure events vs without seizure events. Adapted from: Reaven et al (2018) [54], Piña-Garza et al (2017) [46], Stockl et al (2019) [55], Hollenack et al (2019) [52], Reaven et al (2019) [53] and Strzelczyk et al (2021) [47] .* Costs were converted from Euros to US dollars on 21^st^ June 2022 (exchange rate 1.00 Euro =1.06 US Dollars [xe.com]). ASM, anti-seizure medication; DS, Dravet syndrome; ED, emergency department; LGS, Lennox–Gastaut syndrome; PPPY, per person per year; OP, outpatient; TSC, tuberous sclerosis complex

Total healthcare costs and healthcare resource utilization associated with LGS were substantially higher than matched controls [54], non-LGS epilepsy patients [46], and patients with other DEEs, including DS and TSC [52, 53, 55] (Fig 3A, Additional file 2: Table S1). For example, Reaven et al. [54] reported that, compared to matched controls, annual costs for services were 17 to 20 times higher for patients with possible LGS, and annual costs for drugs were 16 to 38 times higher (Fig 3B). In addition, among LGS patients, costs and healthcare resource utilization were reported to be higher in patients with a seizure event versus those without (Fig 3C) [53], and costs and hospitalizations/ length of stay (LOS) were higher in those prescribed rescue medication vs those not [47] (Additional file 3: Fig S1).

**Fig 4:**
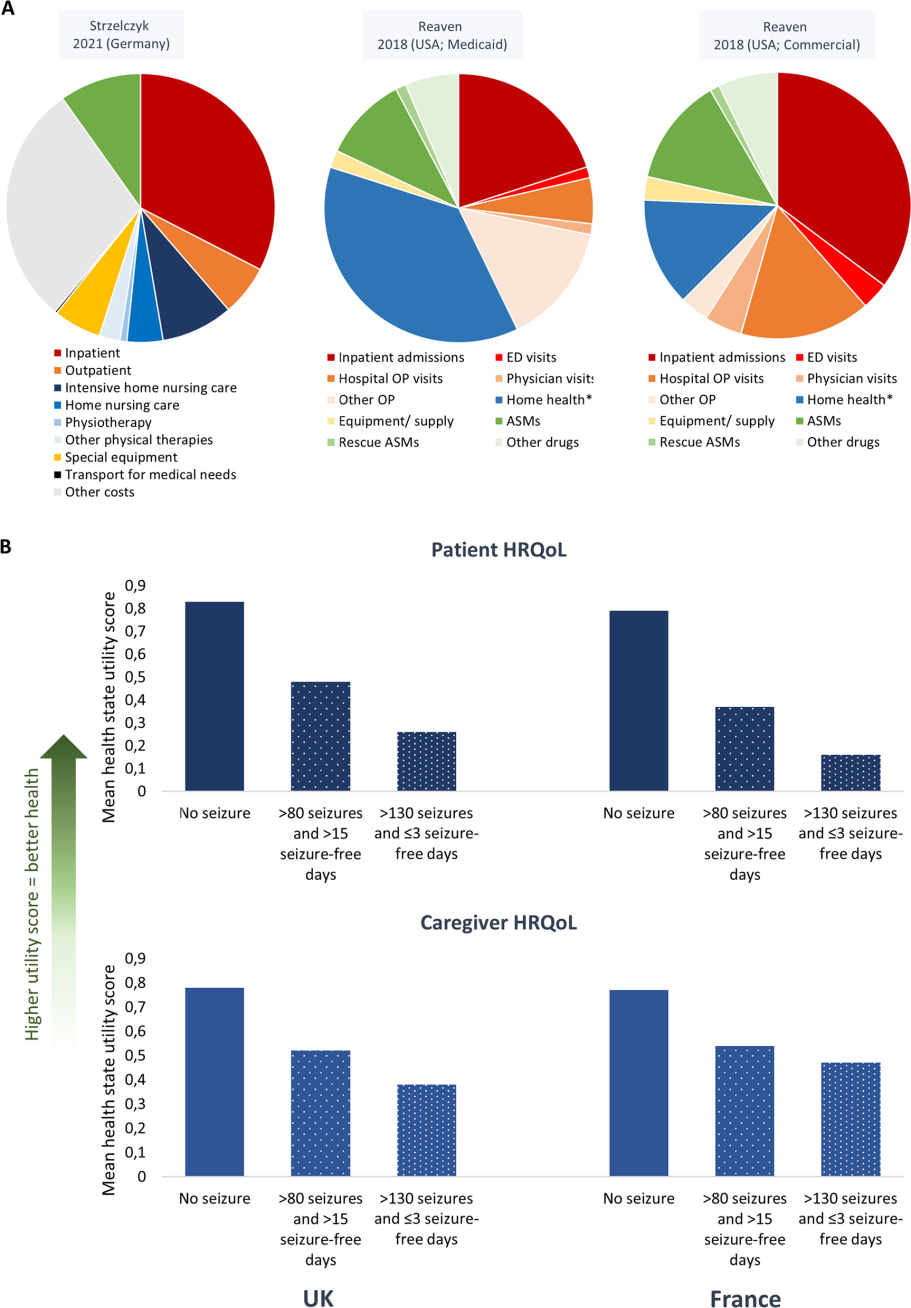
**A** Cost drivers according to inpatient and emergency department admissions (red), outpatient visits (orange), home health (home nursing and physiotherapy; blue), equipment (yellow) and medication (green) costs (where reported) **B** Patient and caregiver HRQoL according to number of drop seizures per month and number of seizure-free days in the UK and France. Mean health state utility scores for a hypothetical patient with LGS and a hypothetical caregiver of a patient with LGS. **A** Adapted from: Strzelczyk et al (2021) [47] and Reaven et al (2018) [54]. Home health= home-based nursing, personal care, and residential habilitation services. **B** Adapted from Auvin et al (2021) [61]. ASM, anti-seizure medication; ED, emergency department; HRQoL, health-related quality of life; OP, outpatient

The studies showed that patients with LGS require a multitude of healthcare resources including general/family practitioner visits, outpatient visits to the neurologist/epilepsy clinic, laboratory and radiology services, medication, physiotherapy/habilitation services and other physical therapies, inpatient and emergency department visits, as well as home-based nursing [40, 47, 51, 53, 54, 56] (Additional file 2: Table S1). Across the studies, inpatient care and home-based care were significant cost drivers, whereas pharmacy costs, particularly for ASMs, generally represented a smaller share of all healthcare costs (Fig 4A, Additional file 2: Table S1) [46, 47, 51–55]. In Germany the greatest contributors to healthcare costs were inpatient care (33%) especially hospital stays related to epilepsy, followed by home nursing care (including intensive home nursing) (13%), and medication (10%) (Fig 4A, Additional file 2: Table S1) [47]. ASMs represented only 14% of the overall medication costs despite their ubiquitous use. Similarly, Piña-Garza et al. [46] reported that the main contributor to healthcare costs was home-based care in children aged 1–18 years (mean costs of $12,396 to $18,360 PPPY across across different pediatric age groups) and long-term care in the 60-year-old cohort ($16,215 PPPY), whereas mean pharmacy costs encompassed a smaller proportion of the costs at $1592–$5630 PPPY across all age groups. In the study by Reaven et al. [54], the largest contributors to the healthcare costs in LGS patients were inpatient care in the commercial-insured cohort and home health services (home-based nursing, personal care, and residential habilitation services) in the medic aid-insured cohort (Fig 4A, Additional file 2: Table S1).

While the costs of ASMs may account for a relatively small proportion of the overall healthcare costs, data from several studies suggest that the burden of ASMs on patients and the overall pharmacy costs is high. For example, in a study based on IBM® MarketScan^®^ medic aid database, the mean number of distinct ASMs during the 12-month study period averaged 2.4, 2.3, and 2.2, in probable LGS, probable DS, and other refractory epilepsies, respectively, accounting for 67.3%, 62.8%, 52.3% of the pharmacy costs [52]. Likewise, using the IBM^®^ MarketScan^®^ commercial database, the mean number of distinct ASMs was 3.4, 2.6, and 2.1, respectively, representing 72.6%, 70.8%, and 65.8% of the pharmacy costs [55]. In addition, Chin et al (2021) reported that in patients with confirmed LGS the mean number of ASMs was 6.7 (SD 3.4) during the 8–12 years follow-up period, with only 3.7% being prescribed only one ASM during this period, while the mean number of ASMs per year was 1.06 during 2010–13 and 1.12 during 2014–17 [40]; this suggests that patients had to switch to different ASMs many times over the course of the study period. Similarly, in the study in Germany, patients generally received 1–3 different ASM combinations each year (range: 1–9) and most patients received 2–4 different ASMs [range: 1–12] over the entire 10-year observable period [47]. Of note, the use of other drugs was also substantially higher in LGS patients than in control populations (Fig 2B) [54]. In both Germany [47] and the UK [40] the most commonly prescribed ASMs were valproate, lamotrigine, clobazam, oxcarbazepine (in Germany) and levetiracetam (in the UK); in the UK rufinamide was prescribed in 27% of patients with confirmed LGS. In the US, Pina-Garza et al. [46] reported LGS patients may be inadequately treated; the majority, but importantly not all probable LGS patients had ≥1 claim for an ASM (62.6–82.3% across age cohorts), while clobazam and rufinamide, both specifically licensed for LGS in the US, were infrequently used, especially in the older cohorts (17.5% for clobazam and 7.4% for rufinamide in patients ≤5 years, 5.5% for both ASMs in the 20-year cohort and <1.0% of patients in the 60-year cohort).

Regarding hospitalizations and LOS, in Germany there was a large variation between patients, with some being hospitalized many times (median hospitalization rate: 1 [range: 0–13 PPPY]) with a median LOS of 3 days (range: 0–804 days) [47]. In a study describing the nature of acute hospitalizations in LGS patients, 46–58% were considered epilepsy-related across health plans (Commercial and Medicaid) compared to 63–70% for DS, 26% for TSC, and 37–51% for other DEEs; 6–7% were pneumonia-related, and approximately 2% were injury-related [56]. Across the different DEEs, admission to the intensive care unit (ICU) accounted for 31% of all hospitalizations, and ICU use was associated with a longer LOS (mean 8.0 [SD 16.8] days vs. 4.0 [SD 7.9] days for non-ICU use). Furthermore, readmission rates were high, with approximately 9–10% of patients readmitted within the month and 42–45% within a year of discharge [56]. Concerning injuries, 67% of LGS patients reported at least one injury over the 10-year study period in Germany [47], while in the study by Reaven et al. [53], 22% (2329 of 10,618) of LGS patients with a medically-treated seizure event across insurance types had an injury.

### Health-related quality of life

Five studies, published between 1993 and 2021, were identified that reported on the HRQoL of patients with LGS and/or their caregivers using qualitative and/or quantitative methods; one study reported on both caregiver and patient perspectives [58], 2 focused on caregivers [59, 60] and 2 on patients [61, 62] (Table 2). Three studies used qualitative methods (one for both patient and caregiver HRQoL, and two for caregiver HRQoL); the quality of the qualitative studies was assessed to be grade I (highest methodological and reporting quality) in one study and grade III (limitations in methodological and reporting quality) in 2 studies (Additional file 2: Table S4). Three studies used quantitative methods (one assessed caregiver HRQoL using the SF-36 and the HADS (Hospital Anxiety and Depression Scale), while two studies used a VAS (visual analog scale). The quality of the quantitative studies was assessed to be grade II (moderate-high methodological and reporting quality) in 2 studies and grade III in one study (Additional file 2: Table S5).

All three studies reporting on the HRQoL of caregivers found that LGS had substantial negative effects. Gallop et al. [58] investigated the HRQoL of parent caregivers (N = 40) both quantitively and qualitatively. Using the SF-36v2, a widely used generic instrument to measure health status, the parents’ mental health summary score (a composite of social functioning, vitality and mental health) was found to be below average (45 for LGS parents vs. 50 for US general population). In addition, 58% of parents experienced anxiety according to the HADS. Using semi-structured interviews with the parents, the authors determined that the parents were impacted physically, emotionally, socially and financially. Gibson [59] similarly reported that LGS had a significant physical, emotional, social and financial impact on parents, while siblings were also affected, frequently taking on a caregiver role themselves early in life. Furthermore, Murray reported in 1993 that parents had to deal with a lot of uncertainty around diagnosis, etiology, seizure activity, treatment and prognosis, which caused feelings of guilt and stress in some parents [60].

Gallop et al. [58] also investigated parents’ perceptions of how LGS impacted their children, describing the profound impact that the syndrome conferred on the daily lives of children with LGS; in particular, physical, social, cognitive and behavioral aspects of their lives were disrupted. Studies by Auvin et al. [61] and Radu et al. [62] used an alternative method of measuring patient HRQoL in a quantitative manner whereby patients and/or caregivers of patients with LGS, DS, or other epilepsies were asked to score the patient HRQoL based on hypothetical vignettes of patients with LGS (or DS) according to how many seizures and seizure-free days the hypothetical patient experienced. These studies have suggested that fewer seizures and additional seizure-free days are associated with better patient HRQoL, with seizure-free days having the greatest consequence on HRQoL (Fig 4B). Of note, this vignette-based method for estimating HRQoL focused specifically on the impact of seizure frequency/seizure-free days as opposed to a more holistic HRQoL assessment. The values were used in the NICE (National Institute for Health and Care Excellence) appraisal in the UK to inform the cost-effectiveness of cannabidiol for the adjuvant treatment of seizures associated with LGS [63].

## Discussion

We conducted a comprehensive SLR following pre-specified inclusion/exclusion criteria to understand the burden of LGS, providing a descriptive summary of the literature while identifying gaps in the current knowledge. The epidemiology studies confirm that LGS is a rare syndrome, whereby the prevalence varied from 4.2 to 60.8 per 100,000 people across studies for probable LGS and 2.9 to 28 per 100,000 people for confirmed/narrow definition of LGS. Annual costs per patient and healthcare resource utilization were substantial across all studies, confirming the high economic burden associated with LGS. In addition, studies showed that the HRQoL of patients and caregivers was adversely affected, and seizure events were associated with higher costs and worse HRQoL. Furthermore, LGS was associated with high mortality rates compared to the general population and those with epilepsy in general.

The burden of illness studies relies heavily on how the disease is defined. However, for LGS there are inherent difficulties in using the currently available source data where there has been no specific code for LGS in the ICD coding in many regions. An extension code for LGS in the ICD-10-CM coding system (G40. 812) has only been available since 2015, and it is only used in some regions. The recent release of ICD-11 includes a specific code for LGS (8A62.1 Lennox–Gastaut syndrome) [64], however, it may be many years before the benefits are seen in real-world research, including the burden of illness studies, which require a sufficient number of patients and observation period. Without a precise definition of LGS and a lack of consistency in using recent and specific diagnostic codes for LGS, there was considerable heterogeneity between studies on how LGS was defined. The retrospective claims analysis studies generally used a “probable LGS” definition that included appropriate ICD-9 or ICD-10 codes for epilepsy and developmental delay and a prescription for a relevant ASM. Piña-Garza et al (2017) developed a machine-learning classification model to identify patients with LGS [46], which was subsequently used and modified for other studies [53, 54, 56]. The methods were latterly reported in more detail by Vekeman et al (2019), showing that an LGS classifier that used input variables including the number of distinct ASMs received, epilepsy-related outpatient/inpatient visits, electroencephalogram procedures and claims for delayed development, showed high sensitivity and specificity in identifying LGS patients [65]. On the other hand, Chin et al (2021) was able to circumvent the limitations of the ICD coding by using the electronic medical records from the CPRD in the UK that included a code for LGS (the confirmed LGS group) [40]. Other smaller retrospective, observational studies reporting on the epidemiology of LGS included patients who had been diagnosed based on standard diagnostic/ILAE criteria [39, 42–45, 48], while the HRQoL studies generally recruited caregivers and/or patients through support groups, patient associations, clinicians and/or websites [58–62]. Other areas of heterogeneity across the studies included the size of the populations, the ages of the patients and the length of the follow-up period. Another limitation is that LGS is likely to be underdiagnosed/ misdiagnosed, especially in adults [11, 14], and therefore many aspects of the burden of illness may have been underestimated.

Many of the studies identified are recently published, particularly those on costs and healthcare resources, which may reflect the development of new treatment options, whereby the data can be used to inform payers and health technology assessment agencies. We did not look at the impact of interventions on our outcomes. However, compared to standard of care, studies have estimated that rufinamide is a cost-effective treatment [66, 67], while cannabidiol was found to be cost-effective in a model that was based on absolute seizure frequency and seizure-free days and took account of the impact of caregivers’ burden [68], but in a different model, it was determined not to be cost-effective at a willingness-to-pay threshold of $150,000/ quality-adjusted life-year [69]. As with DS [70–73], studies have suggested that seizure events in LGS are associated with higher costs and worse HRQoL, and therefore it is hoped that current and future improvements in the treatment will help to alleviate some of the burdens on healthcare resources and costs and improve the HRQoL of patients and caregivers.

We found only a few studies evaluating the HRQoL in patients and caregivers. In a recent SLR describing the impact of a range of DEEs (including LGS) on the HRQoL of caregivers and the wider family unit Gallop et al. [58] identified only one study of sufficient quality regarding caregivers of patients with LGS (also identified hererin) [38]. However, across the DEEs, the authors found that most of the studies reported a negative impact on the HRQoL of caregivers, and they highlighted the challenges of evaluating caregiver HRQoL, especially using generic instruments, because parents of young children with complex chronic diseases adapt and become habituated to their decrease in quality of life [38]. The same can apply to patients, although measuring HRQoL in patients with LGS generally requires a parent proxy because of the patient’s young age and/or cognitive dysfunction, as well as not knowing a time before their condition with which to compare to.

We identified no studies reporting on indirect costs, and therefore it is likely that the overall economic burden has been underestimated due to studies only reporting on direct costs. Indeed, the financial burden among patients and caregivers has also been little studied across other DEEs including DS and TSC [25]. However, the few studies that are available on DS caregivers and TSC patients or caregivers have shown that indirect costs are relevant [72, 74–76], with a particular impact on mothers having to reduce their working hours or stop working completely [72, 74, 77]. Overall, evaluating the caregiver burden including indirect costs, productivity losses and HRQoL requires complex, preferably prospective, studies that may have been hindered by a lack of having large patient advocacy groups from which to recruit participants, as well as a lack of priority for health research funding, among other factors.

### Strengths and limitations

As far as we are aware this is the first SLR on the burden of illness in LGS. The SLR has several strengths in that it was a comprehensive review performed according to the PRISMA guidelines, with no limitations on language or date (except for abstracts), whereby the search terms were developed by an experienced information scientist.

The main limitations have been discussed above, including the challenges around identifying the LGS population and the paucity of studies, especially on caregiver HRQoL and indirect costs. Furthermore, many of the studies, especially regarding costs and resources, were conducted in the USA, with limited evidence in Europe, and no evidence from other parts of the world despite not having any language restrictions. This country-bias may reflect the lack of research funding as described above, with perhaps some publication bias against non-native English speaking countries [78, 79]. The situation in low and middle income countries is especially complex and many aspects of the burden of illness, especially mortality, costs, and resources are likely to be very different from high income countries. For example, a study of the global burden of epilepsy (among other conditions) reported that 81% of epilepsy-deaths occurred in low and middle countries [80], reflecting both the larger number of people with epilepsy and the limited resources with which to effectively manage and treat patients [81]. Overall, there is an imperative need for innovative solutions to improve the outcomes of patients in these countries [82, 83].

We restricted the search specifically to LGS, excluding studies which encompassed refractory epilepsy in general, even if they had a few LGS cases within a more general population of refractory epilepsy patients, which may have limited our evidence base, especially as LGS can evolve from or be part of other epilepsy syndromes, e.g. TSC [84]. In addition, while the literature was searched in major electronic databases, we may still have missed some studies from other databases or from the grey literature. We did not identify any extra relevant studies when we searched the reference lists of other SLRs, reviews and economic evaluations suggesting that our methods, including the search terms and databases used, were robust.

Future research directions

In this SLR we have found that there is lack of studies reporting on indirect costs, and patient and caregiver HRQoL. These are extremely important and relevant outcomes for this complex syndrome that goes beyond seizures. In addition, there is a lack of studies on all aspects of the burden of illness in low and middle income countries, and more research is needed in these areas, possibly through enhanced collaboration with established research groups in high income countries and the pharmaceutical industry. Patient advocacy groups for LGS should be empowered across all countries, perhaps through the use of virtual events and an increase in on-line resources and communities.

Studies in this SLR have reported that seizure events are associated with higher resource and costs, and lower patient HRQoL. Cannabidiol and fenfluramine are the most recently approved therapies for the treatment of seizures associated with LGS (Fig 1). ASMs in clinical development include soticlestat and carisbamate. Soticlestat has recently successfully completed Phase 2 clinical deveopment as an adjunctive therapy in pediatric patients with DS or LGS [85], with Phase 3 trials underway (NCT04938427 and NCT05163314). A Phase 3 clinical trial assessing carisbamate in patients with LGS has also been initiated (NCT05219617). It will certainly be of interest in the future to determine the impact that these potential and new ASMs [89] have on the burden of illness in LGS, especially patient and caregiver HRQoL. Of concern though is the apparent lack of other therapies in development for patients with LGS at present.

Also of note is that LGS has multiple and varied etiologies, and with advances in neuroimaging and genetics the conditions within this syndrome are now largely defined and named by their etiology. These etiologies are a more precise guide to prognosis and management than the designation of LGS. William Lennox’s views on eugenics [87, 88] have resulted in his name being removed from named lectures in the USA. The International League Against Epilepsy (ILAE) decided to keep the term Lennox–Gastaut in the 2022 ILAE Syndromes Definitions arguing that this was because medications are licensed for LGS and that a better collective name had not been coined for DEE with slow spike and wave and tonic seizures in childhood [86]. However, the term Lennox–Gastaut may gradually be retired in the next decade.

## Conclusions

LGS is associated with a substantial burden of illness, with seizure events associated with higher costs and worse HRQoL. However, with no ICD-10 coding for LGS available in many regions, several studies had to rely upon indirect methods to identify their patient populations. More research is needed, especially in evaluating the caregiver burden, including HRQoL, productivity losses and indirect costs, where there is a notable lack of studies.

## Supplementary Information

**Additional file 1**. Search terms.

**Additional file 2**. **Supplementary Table S1:** Healthcare resource utilization and costs in patients with LGS; **Table S2:** Quality assessment checklist for prevalence studies; **Table S3:** Quality assessment checklist of cost-of-illness studies; **Table S4:** Quality assessment checklist for qualitative HRQoL studies; **Table S5:** HRQoL: Quality assessment checklist for quantitative HRQoL studies.

**Additional file 3**. **Supplementary Figure S1:** Costs, annual hospitalization rate and length of stay (LOS) in LGS patients in years where patients were prescribed with rescue medication vs years where patients were not prescribed rescue medication.

## Data Availability

The datasets used and/or analyzed during the current study are available from the corresponding author on reasonable request.

## Abbreviations

ASMs: Anti-seizure medications
CI: Confidence interval
CPRD: Clinical Practice Research Datalink
DEE: Developmental and epileptic encephalopathy
DS: Dravet syndrome
EEG: Electroencephalogram
HADS: Hospital Anxiety and Depression Scale
HRQoL: Health-related quality of life
ICU: Intensive care unit
LGS: Lennox–Gastaut syndrome
LOS: Length of stay
MADDS: Metropolitan Atlanta Developmental Disabilities Study
NICE: National Institute for Health and Care Excellence
ONS: Office of National Statistics
PICOS: population, intervention, comparator, outcomes and study design
PRISMA: Preferred reporting items for systematic reviews and meta-analyses
PPPY: Per person per year
SD: Standard deviation
SLR: Systematic literature review
TSC: Tuberous sclerosis complex
