# Supplementary material for: The burden of illness in Lennox–Gastaut syndrome: a systematic literature review
Source: Orphanet J Rare Dis. 2023 Mar 1;18:42. doi: 10.1186/s13023-023-02626-4 (PMC9979426; doi:10.1186/s13023-023-02626-4)
Supplement: Supplementary file 1 — Additional file 1. Search terms. [file 13023_2023_2626_MOESM1_ESM.pdf]

The burden of illness in Lennox-Gastaut syndrome: a systematic literature review: search terms

Conceptual search purpose: The purpose of this search to identify the evidence associated with the burden of illness in individuals with Lennox-Gastaut syndrome. The search strategy is focused on identifying epidemiolocal data (prevalence/incidence), resource use (costs and economic analyses) and disease burden (health related quality of life).

Database: MEDALL
Host: Ovid
Data parameters: 1946 to September 09, 2021
Date of search: 10 September 2021

Search strategy	Search Narrative
1 Lennox Gastaut Syndrome/ (393)<br>2 ((Lennox$ adj3 Gastaut$) or "lennox$ syndrom$" or "petit mal variant").ti,ab,kw,kf. (1599)<br>3 1 or 2 (1699)	This cluster of terms represents the condition under review, namely: Lennox Gastaut Syndrome.<br><br>Line 1 is the controlled indexing heading for Lennox Gastaut Syndrome (MeSH indexing indicated by /).<br><br>Line 2 is a free-text search line. This uses a proximity marker (adj3) meaning that Lennox is searched within two words of Gastaut and in any order (e.g. Lennox WORD WORD Gastaut OR Gastaut WORD WORD Lennox). Truncation (indicated by $) is used to identify alternate word endings such Gastaut or Gastaut's syndrome.<br><br>The free-text lines are searched in the following fields:<br><br>• ti – title;<br>• ab = abstract;<br>• kw = keyword;<br>• kf = author key field (keywords chosen by authors).<br><br>The condition terms were compared to existing systematic reviews to check the currency of search terms.[7,8] No modifications were identified following this review.
4 Epidemiologic Studies/ (8796)<br>5 incidence/ or prevalence/ (570714)<br>6 (Epidemiolo$ or prevalenc$ or incidenc$).ti,ab,kw,kf. (1777396)<br>7 4 or 5 or 6 (1935263)	This cluster of terms represents a search for prevalence or incidence. The cluster is based on the search filter developed by Royle and Waugh for epidemiology studies.[1]
8 exp economics/ (626495)<br>9 exp "Costs and Cost Analysis"/ (248953)<br>10 exp Economics, Hospital/ or Financial management, hospital/ (32555)<br>11 Economics, Medical/ (9151)	This cluster of terms represents the search for resource use. The search filter is based on the NHS CRD EED filter and it has been enhanced by comparison with the CADTH economic evaluations/costs/economic models filter.[2,4]

12 economics, nursing/ (4006)<br>13 economics, pharmaceutical/ (3016)<br>14 (economic* or cost or costs or costly or costing or expense or expenses or price or prices or pricing or pharmacoeconomic* or expense or expenses or CEA or CUA or CBA or CMA).ti,ab,kw,kf. (975213)<br>15 (resource*1 and (allocation or utili* or using or usage or use*1)).ti,ab,kw,kf. (257393)<br>16 (expenditure* not energy).ti,ab,kw,kf. (33031)<br>17 (value adj1 (money or monetary)).ti,ab,kw,kf. (743)<br>18 (budget* or fiscal or funding or financial or finance*).ti,ab,kw,kf. (199155)<br>19 (decision* adj2 (tree* or analy* or model*)).ti,ab,kw,kf. (28696)<br>20 (markov or monte carlo).ti,ab,kw,kf. (73020)<br>21 ((statistical or simulation$) adj2 model$).ti,ab,kw,kf. (50200)<br>22 8 or 9 or 10 or 11 or 12 or 13 or 14 or 15 or 16 or 17 or 18 or 19 or 20 or 21 (1790163)	
23 ("Quality of Life in Childhood Epilepsy" or QOLCE).ti,ab,kw,kf. (88)<br>24 ("Quality of Life in Epilepsy for Adolescents" or "QOLIE-AD-48").ti,ab,kw,kf. (27)<br>25 ("The Impact of Childhood Illness Scale" or ICI).ti,ab,kw,kf. (11131)<br>26 ("The Hague Restrictions in Childhood Epilepsy Scale" or HARCES).ti,ab,kw,kf. (6)<br>27 ("Quality of Life in Epilepsy Inventory for Adolescents" or "QoLIE-AD-48").ti,ab,kw,kf. (27)<br>28 ("Quality of Life in Pediatric Epilepsy" or QoLPES).ti,ab,kw,kf. (9)<br>29 ("Quality of Life in Childhood Epilepsy" or (QoLCE or "G-QoLCE")).ti,ab,kw,kf. (88)<br>30 ("Impact of Pediatric Epilepsy Scale" or IPES).ti,ab,kw,kf. (83)<br>31 ("Health-Related Quality of Life Measure for Children with Epilepsy" or "CHEQoL-25").ti,ab,kw,kf. (17)<br>32 DISABKIDS.ti,ab,kw,kf. (103)<br>33 ("Epilepsy and Learning Disability Quality of Life" or ELDQoL).ti,ab,kw,kf. (8)<br>34 ("Glasgow Epilepsy Outcome Scale" or "GEOS-YP").ti,ab,kw,kf. (7)<br>35 "Impact of Epilepsy Schedule".ti,ab,kw,kf. (1)<br>36 (QOLIE or QOLIE-31 or QOLIE-10).ti,ab,kw,kf. (552)<br>37 "Pediatric Quality of Life Inventory or PedsQL".ti,ab,kw,kf. (0)	This cluster of terms represents the search for disease burden and health-related quality of life. The filter has been conceived in two parts:<br><br>i) lines 23-37 represents condition specific instruments. These were developed from an informal scoping of intervention studies, reference to a recent review,[9] and the clinical knowledge of the authors.<br><br>ii) lines 26-51 is based on Paisley and Booth's HRQoL filter, which has been compared to CADTH's filter for Health Utilities, and adapted through development by the information specialist undertaking the searches.[3,4]<br><br>These two parts are combined at line 67 using the Boolean connector OR, meaning that the concepts unique to both parts will be returned.

38 23 or 24 or 25 or 26 or 27 or 28 or 29 or 30 or 31 or 32 or 33 or 34 or 35 or 36 or 37 (11982)	
39 (15D or 15-D or 15 dimension).ti,ab,kw,kf,ot,hw. (5649)	
40 (eq-5d or eq5d or eq-5 or eq5 or EQ-5D-Y or euro qual or euroqual or euro qual5d or euroqual5d or euro qol or euroqol or euro qol5d or euroqol5d or euro quol or euroquol or euro quol5d or euroquol5d or eur qol or eurqol or eur qol5d or eur qol5d or eur?qul or eur?qul5d or euro$ quality of life or european qol or EQ-5D-3L).ti,ab,ot,hw,kw. (13675)	
41 (sf6 or sf 6 or SF-6D or short form 6 or short-form 6 or short-form six or shortform 6 or sf six or sfsix or shortform six or short form six).ti,ab,ot,hw,kw. (3052)	
42 (sf10 or sf 10 or short form 10 or short-form 10 or short-form ten or shortform 10 or sf ten or sften or shortform ten or short form ten).ti,ab,ot,hw,kw. (143)	
43 (sf12 or sf 12 or short form 12 or short-form 12 or short-form twelve or shortform 12 or sf twelve of sftwelve or shortform twelve or short form twelve).ti,ab,ot,hw,kw. (6562)	
44 (sf16 or sf 16 or short form 16 or short-form 16 or short-form sixteen or shortform 16 or sf sixteen or sfsixteen or shortform sixteen or short form sixteen).ti,ab,ot,hw,kw. (35)	
45 (sf20 or sf 20 or short form 20 or short-form 20 or short-form twenty or shortform 20 or sf twenty of sftwenty or shortform twenty of short form twenty).ti,ab,ot,hw,kw. (418)	
46 (sf36 or sf 36 or short form 36 or short-form 36 or short-form thirty six or shortform 36 or sf thirtysix or sf thirty six or shortform thirstysix or shortform thirty six or short form thirty six or short form thirtysix or short form thirty six).ti,ab,ot,hw,kw. (27662)	
47 (health utilities index$ or (hui or hui1 or hui2 or hui3 or hui4 or hui-4 or hui-1 or hui-2 or hui-3)).ti,ab,ot,hw,kw. (1983)	

48 ("time trade off" or "time tradeoff" or "time trade-off" or TTO).ti,ab,ot,hw,kw. (2028)<br>49 (standard gamble$ or SG).ti,ab,ot,hw,kw. (12070)<br>50 ("discrete choice" or DCE).ti,ab,ot,hw,kw. (8142)<br>51 (AQoL or "Assessment of Quality of Life").ti,ab,ot,hw,kw. (2067)<br>52 Quality-Adjusted Life Years/ (13713)<br>53 (HRQoL or HRQL or HQL or QoL or (quality adj3 life) or HYE or HYES or (health$ adj3 year$)).ti,ab,ot,hw,kw.<br>(416018)<br>54 quality of life/ (220474)<br>55 value of life/ (5757)<br>56 uncertainty/ (14495)<br>57 (uncertain$ or wellbeing or "well being" or rosser or "willingness to pay").tw. (296805)<br>58 (utilit$ or disutility$).ti,ab,kw,kf. (231234)<br>59 (illness state$1 or health state$ or health status or Quality adjusted life year$ or QALY or QALD or qale or<br>qtime or life year$ or ICER or "incremental cost").ti,ab,ot,hw,kw. (197462)<br>60 (burden and (disease or illness or caregiver or home)).tw. (107163)<br>61 (lost adj2 (productivity or work or employment or earnings)).ti,ab,kw,kf. (3131)<br>62 (((disability or diseas$) adj3 adjust$) or daly$).ti,ab,kw,kf. (10374)<br>63 (preference* adj3 (valu* or measur* or health or life or estimat* or elicit* or disease or score* or instrument<br>or instruments)).ti,ab,kf,kw. (12277)<br>64 (self report$ or (patient adj report$ adj outcome$)).ti,ab,kw,kf. (198747)<br>65 (mortality or death).ti,ab,kw,kf. (1478283)<br>66 39 or 40 or 41 or 42 or 43 or 44 or 45 or 46 or 47 or 48 or 49 or 50 or 51 or 52 or 53 or 54 or 55 or 56 or 57 or<br>58 or 59 or 60 or 61 or 62 or 63 or 64 or 65 (2665328)<br>67 38 or 66 (2674844)	
68 7 or 22 or 67 (5538580)<br>69 3 and 68 (323)	Line 68 combines the three concepts of this search, namely:<br><br>• Line 7 (epidemiology)<br>• Line 22 (resource use)

	• Line 67 (burden of disease/HRQoL)<br><br>The Boolean connector OR is used meaning that all concepts are combined.<br><br>Line 69 combines the condition terms (line 3) AND the search filters to complete the search in MEDLINE.

Database: Embase (without conferences)
Host: Ovid
Data parameters: 1980 to 2021 Week 35
Date of search: 10 September 2021

#	Searches	Results
1	*Lennox Gastaut Syndrome/	1424
2	((Lennox$ adj3 Gastaut$) or "lennox$ syndrom$" or "petit mal variant").ti,ab,kw.	2544
3	1 or 2	2974
4	incidence/	465725
5	prevalence/	798135
6	(Epidemiolo$ or prevalenc$ or incidenc$).ti,ab,kw.	2409155
7	4 or 5 or 6	2659622
8	exp economic evaluation/	320231
9	health-economics/	29645
10	exp health-care-cost/	304430
11	exp pharmacoeconomics/	207621
12	(economic* or cost or costs or costly or costing or expense or expenses or price or prices or pricing or pharmacoeconomic* or expense or expenses or CEA or CUA or CBA or CMA).ti,ab,kw.	1231979
13	(resource*1 and (allocation or utili* or using or usage or use*1)).ti,ab,kw.	337470
14	(expenditure* not energy).ti,ab,kw.	43745
15	(value adj1 (money or monetary)).ti,ab,kw.	939
16	(budget* or fiscal or funding or financial or finance*).ti,ab,kw.	270670
17	(decision* adj2 (tree* or analy* or model*)).ti,ab,kw.	39671
18	(markov or monte carlo).ti,ab,kw.	81378
19	((statistical or simulation$) adj2 model$).ti,ab,kw.	60619
20	8 or 9 or 10 or 11 or 12 or 13 or 14 or 15 or 16 or 17 or 18 or 19	2105339
21	("Quality of Life in Childhood Epilepsy" or QOLCE).ti,ab,kw.	134
22	("Quality of Life in Epilepsy for Adolescents" or "QOLIE-AD-48").ti,ab,kw.	45
23	("The Impact of Childhood Illness Scale" or ICI).ti,ab,kw.	14607
24	("The Hague Restrictions in Childhood Epilepsy Scale" or HARCES).ti,ab,kw.	11
25	("Quality of Life in Epilepsy Inventory for Adolescents" or "QoLIE-AD-48").ti,ab,kw.	45

26	("Quality of Life in Pediatric Epilepsy" or QoLPES).ti,ab,kw.	12
27	("Quality of Life in Childhood Epilepsy" or (QoLCE or "G-QoLCE")).ti,ab,kw.	134
28	("Impact of Pediatric Epilepsy Scale" or IPES).ti,ab,kw.	115
29	("Health-Related Quality of Life Measure for Children with Epilepsy" or "CHEQoL-25").ti,ab,kw.	25
30	DISABKIDS.ti,ab,kw.	178
31	("Epilepsy and Learning Disability Quality of Life" or ELDQoL).ti,ab,kw.	13
32	("Glasgow Epilepsy Outcome Scale" or "GEOS-YP").ti,ab,kw.	9
33	"Impact of Epilepsy Schedule".ti,ab,kw.	1
34	(QOLIE or QOLIE-31 or QOLIE-10).ti,ab,kw.	1092
35	"Pediatric Quality of Life Inventory or PedsQL".ti,ab,kw.	0
36	21 or 22 or 23 or 24 or 25 or 26 or 27 or 28 or 29 or 30 or 31 or 32 or 33 or 34 or 35	16162
37	(15D or 15-D or 15 dimension).ti,ab,kw.	7059
38	(eq-5d or eq5d or eq-5 or eq5 or EQ-5D-Y or euro qual or euroqual or euro qual5d or euroqual5d or euro qol or euroqol or euro qol5d or euroqol5d or euro quol or euroquol or euro quol5d or euroquol5d or eur qol or eurqol or eur qol5d or eur qol5d or eur?qul or eur?qul5d or euro$ quality of life or european qol or EQ-5D-3L).ti,ab,ot,hw,kw.	26595
39	(sf6 or sf 6 or SF-6D or short form 6 or short-form 6 or short-form six or shortform 6 or sf six or sfsix or shortform six or short form six).ti,ab,ot,hw,kw.	3949
40	(sf10 or sf 10 or short form 10 or short-form 10 or short-form ten or shortform 10 or sf ten or sften or shortform ten or short form ten).ti,ab,ot,hw,kw.	224
41	(sf12 or sf 12 or short form 12 or short-form 12 or short-form twelve or shortform 12 or sf twelve of sftwelve or shortform twelve or short form twelve).ti,ab,ot,hw,kw.	12504
42	(sf16 or sf 16 or short form 16 or short-form 16 or short-form sixteen or shortform 16 or sf sixteen or sfsixteen or shortform sixteen or short form sixteen).ti,ab,ot,hw,kw.	65
43	(sf20 or sf 20 or short form 20 or short-form 20 or short-form twenty or shortform 20 or sf twenty of sftwenty or shortform twenty of short form twenty).ti,ab,ot,hw,kw.	517
44	(sf36 or sf 36 or short form 36 or short-form 36 or short-form thirty six or shortform 36 or sf thirtysix or sf thirty six or shortform thirstysix or shortform thirty six or short form thirty six or short form thirtysix or short form thirty six).ti,ab,ot,hw,kw.	52721
45	(health utilities index$ or (hui or hui1 or hui2 or hui3 or hui4 or hui-4 or hui-1 or hui-2 or hui-3)).ti,ab,ot,hw,kw.	3744
46	("time trade off" or "time tradeoff" or "time trade-off" or TTO).ti,ab,ot,hw,kw.	3028
47	(standard gamble$ or SG).ti,ab,ot,hw,kw.	17941
48	("discrete choice" or DCE).ti,ab,ot,hw,kw.	12285
49	(AQoL or "Assessment of Quality of Life").ti,ab,ot,hw,kw.	3383
50	Quality-Adjusted Life Years/	29703

51	(HRQoL or HRQL or HQL or QoL or (quality adj3 life) or HYE or HYES or (health$ adj3 year$)).ti,ab,ot,hw,kw.	719594
52	"quality of life"/	520565
53	socioeconomics/	142119
54	uncertainty/	37234
55	(uncertain$ or wellbeing or "well being" or rosser or "willingness to pay").tw.	377239
56	(utilit$ or disutility$).ti,ab,kw.	319447
57	(illness state$1 or health state$ or health status or Quality adjusted life year$ or QALY or QALD or qale or qtime or life year$ or ICER or "incremental cost").ti,ab,ot,hw,kw.	223480
58	(burden and (disease or illness or caregiver or home)).tw.	175208
59	(lost adj2 (productivity or work or employment or earnings)).ti,ab,kw.	4554
60	(((disability or diseas$) adj3 adjust$) or daly$).ti,ab,kw.	14646
61	(preference* adj3 (valu* or measur* or health or life or estimat* or elicit* or disease or score* or instrument or instruments)).ti,ab,kw.	15912
62	(self report$ or (patient adj report$ adj outcome$)).ti,ab,kw.	268351
63	(mortality or death).ti,ab,kw.	2073283
64	37 or 38 or 39 or 40 or 41 or 42 or 43 or 44 or 45 or 46 or 47 or 48 or 49 or 50 or 51 or 52 or 53 or 54 or 55 or 56 or 57 or 58 or 59 or 60 or 61 or 62 or 63	3866328
65	36 or 64	3878406
66	7 or 20 or 65	7318863
67	3 and 66	655
68	(conference abstract or conference review or Conference paper).pt.	4939746
69	67 not 68	438
70	limit 69 to embase	409

Database: APA PsycInfo
Host: Ovid
Data parameters: 1806 to September Week 1 2021
Date of search: 10 September 2021

#	Searches	Results
1	Lennox Gastaut Syndrome/	118
2	((Lennox$ adj3 Gastaut$) or "lennox$ syndrom$" or "petit mal variant").ti,ab.	326
3	1 or 2	335
4	incidence/	0
5	prevalence/	0
6	(Epidemiolo$ or prevalenc$ or incidenc$).ti,ab.	203926
7	4 or 5 or 6	203926
8	exp economic evaluation/	0
9	health-economics/	1021
10	exp health-care-cost/	23522
11	exp pharmacoeconomics/	282

12	(economic* or cost or costs or costly or costing or expense or expenses or price or prices or pricing or pharmacoeconomic* or expense or expenses or CEA or CUA or CBA or CMA).ti,ab.	237930
13	(resource*1 and (allocation or utili* or using or usage or use*1)).ti,ab.	102603
14	(expenditure* not energy).ti,ab.	8651
15	(value adj1 (money or monetary)).ti,ab.	465
16	(budget* or fiscal or funding or financial or finance*).ti,ab.	84803
17	(decision* adj2 (tree* or analy* or model*)).ti,ab.	9427
18	(markov or monte carlo).ti,ab.	7981
19	((statistical or simulation$) adj2 model$).ti,ab.	8078
20	8 or 9 or 10 or 11 or 12 or 13 or 14 or 15 or 16 or 17 or 18 or 19	416669
21	("Quality of Life in Childhood Epilepsy" or QOLCE).ti,ab.	51
22	("Quality of Life in Epilepsy for Adolescents" or "QOLIE-AD-48").ti,ab.	19
23	("The Impact of Childhood Illness Scale" or ICI).ti,ab.	1137
24	("The Hague Restrictions in Childhood Epilepsy Scale" or HARCES).ti,ab.	5
25	("Quality of Life in Epilepsy Inventory for Adolescents" or "QoLIE-AD-48").ti,ab.	18
26	("Quality of Life in Pediatric Epilepsy" or QoLPES).ti,ab.	10
27	("Quality of Life in Childhood Epilepsy" or (QoLCE or "G-QoLCE")).ti,ab.	51
28	("Impact of Pediatric Epilepsy Scale" or IPES).ti,ab.	31
29	("Health-Related Quality of Life Measure for Children with Epilepsy" or "CHEQoL-25").ti,ab.	14
30	DISABKIDS.ti,ab.	43
31	("Epilepsy and Learning Disability Quality of Life" or ELDQoL).ti,ab.	5
32	("Glasgow Epilepsy Outcome Scale" or "GEOS-YP").ti,ab.	6
33	"Impact of Epilepsy Schedule".ti,ab.	0
34	(QOLIE or QOLIE-31 or QOLIE-10).ti,ab.	328
35	"Pediatric Quality of Life Inventory or PedsQL".ti,ab.	0
36	21 or 22 or 23 or 24 or 25 or 26 or 27 or 28 or 29 or 30 or 31 or 32 or 33 or 34 or 35	1615
37	(15D or 15-D or 15 dimension).ti,ab.	272
38	(eq-5d or eq5d or eq-5 or eq5 or EQ-5D-Y or euro qual or euroqual or euro qual5d or euroqual5d or euro qol or euroqol or euro qol5d or euroqol5d or euro quol or euroquol or euro quol5d or euroquol5d or eur qol or eurqol or eur qol5d or eur qol5d or eur?qul or eur?qul5d or euro$ quality of life or european qol or EQ-5D-3L).ti,ab.	2635
39	(sf6 or sf 6 or SF-6D or short form 6 or short-form 6 or short-form six or shortform 6 or sf six or sfsix or shortform six or short form six).ti,ab.	347
40	(sf10 or sf 10 or short form 10 or short-form 10 or short-form ten or shortform 10 or sf ten or sften or shortform ten or short form ten).ti,ab.	20
41	(sf12 or sf 12 or short form 12 or short-form 12 or short-form twelve or shortform 12 or sf twelve of sftwelve or shortform twelve or short form twelve).ti,ab.	1552

42	(sf16 or sf 16 or short form 16 or short-form 16 or short-form sixteen or shortform 16 or sf sixteen or sfsixteen or shortform sixteen or short form sixteen).ti,ab.	4
43	(sf20 or sf 20 or short form 20 or short-form 20 or short-form twenty or shortform 20 or sf twenty of sftwenty or shortform twenty of short form twenty).ti,ab.	61
44	(sf36 or sf 36 or short form 36 or short-form 36 or short-form thirty six or shortform 36 or sf thirtysix or sf thirty six or shortform thirstysix or shortform thirty six or short form thirty six or short form thirtysix or short form thirty six).ti,ab.	5566
45	(health utilities index$ or (hui or hui1 or hui2 or hui3 or hui4 or hui-4 or hui-1 or hui-2 or hui-3)).ti,ab.	687
46	("time trade off" or "time tradeoff" or "time trade-off" or TTO).ti,ab.	428
47	(standard gamble$ or SG).ti,ab.	1071
48	("discrete choice" or DCE).ti,ab.	1252
49	(AQoL or "Assessment of Quality of Life").ti,ab.	593
50	Quality-Adjusted Life Years/	0
51	(HRQoL or HRQL or HQL or QoL or (quality adj3 life) or HYE or HYES or (health$ adj3 year$)).ti,ab.	81246
52	"quality of life"/	43216
53	socioeconomics/	0
54	uncertainty/	9685
55	(uncertain$ or wellbeing or "well being" or rosser or "willingness to pay").tw.	156067
56	(utilit$ or disutility$).ti,ab.	63622
57	(illness state$1 or health state$ or health status or Quality adjusted life year$ or QALY or QALD or qale or qtime or life year$ or ICER or "incremental cost").ti,ab.	23031
58	(burden and (disease or illness or caregiver or home)).tw.	20052
59	(lost adj2 (productivity or work or employment or earnings)).ti,ab.	856
60	(((disability or diseas$) adj3 adjust$) or daly$).ti,ab.	2023
61	(preference* adj3 (valu* or measur* or health or life or estimat* or elicit* or disease or score* or instrument or instruments)).ti,ab.	8210
62	[(self report$ or (patient adj report$ adj outcome$)).ti,ab,kw.]	0
63	(mortality or death).ti,ab.	117895
64	37 or 38 or 39 or 40 or 41 or 42 or 43 or 44 or 45 or 46 or 47 or 48 or 49 or 50 or 51 or 52 or 53 or 54 or 55 or 56 or 57 or 58 or 59 or 60 or 61 or 62 or 63	435194
65	36 or 64	436260
66	7 or 20 or 65	941439
67	3 and 66	77

Database: Cochrane CDSR
Host: Wiley
Data parameters: Issue 9 of 12, September 2021

Date of search: 10 September 2021

ID Search Hits
#1 MeSH descriptor: [Lennox Gastaut Syndrome] this term only 33
#2 ((Lennox* Gastaut*) or "lennox* syndrom*" or "petit mal variant"):ti,ab,kw 271
#3 #1 or #2 271

NB: search retrieved 271 across the Cochrane Library platform. Three (3) of these results were from CDSR, which were downloaded.

Database: Epistemonikos
Host: https://www.epistemonikos.org/
Date of search: 10 September 2021

Lennox Gastaut Syndrome

Database: Embase (limited to conferences 2018-current)
Host: Ovid
Data parameters: 1980 to 2021 Week 35
Date of search: 10 September 2021

#	Searches	Results
1	*Lennox Gastaut Syndrome/	1424
2	((Lennox$ adj3 Gastaut$) or "lennox$ syndrom$" or "petit mal variant").ti,ab,kw.	2544
3	1 or 2	2974
4	incidence/	465725
5	prevalence/	798135
6	(Epidemiolo$ or prevalenc$ or incidenc$).ti,ab,kw.	2409155
7	4 or 5 or 6	2659622
8	exp economic evaluation/	320231
9	health-economics/	29645
10	exp health-care-cost/	304430
11	exp pharmacoeconomics/	207621
12	(economic* or cost or costs or costly or costing or expense or expenses or price or prices or pricing or pharmacoeconomic* or expense or expenses or CEA or CUA or CBA or CMA).ti,ab,kw.	1231979
13	(resource*1 and (allocation or utili* or using or usage or use*1)).ti,ab,kw.	337470
14	(expenditure* not energy).ti,ab,kw.	43745
15	(value adj1 (money or monetary)).ti,ab,kw.	939
16	(budget* or fiscal or funding or financial or finance*).ti,ab,kw.	270670
17	(decision* adj2 (tree* or analy* or model*)).ti,ab,kw.	39671
18	(markov or monte carlo).ti,ab,kw.	81378
19	((statistical or simulation$) adj2 model$).ti,ab,kw.	60619
20	8 or 9 or 10 or 11 or 12 or 13 or 14 or 15 or 16 or 17 or 18 or 19	2105339

21	("Quality of Life in Childhood Epilepsy" or QOLCE).ti,ab,kw.	134
22	("Quality of Life in Epilepsy for Adolescents" or "QOLIE-AD-48").ti,ab,kw.	45
23	("The Impact of Childhood Illness Scale" or ICI).ti,ab,kw.	14607
24	("The Hague Restrictions in Childhood Epilepsy Scale" or HARCES).ti,ab,kw.	11
25	("Quality of Life in Epilepsy Inventory for Adolescents" or "QoLIE-AD-48").ti,ab,kw.	45
26	("Quality of Life in Pediatric Epilepsy" or QoLPES).ti,ab,kw.	12
27	("Quality of Life in Childhood Epilepsy" or (QoLCE or "G-QoLCE")).ti,ab,kw.	134
28	("Impact of Pediatric Epilepsy Scale" or IPES).ti,ab,kw.	115
29	("Health-Related Quality of Life Measure for Children with Epilepsy" or "CHEQoL-25").ti,ab,kw.	25
30	DISABKIDS.ti,ab,kw.	178
31	("Epilepsy and Learning Disability Quality of Life" or ELDQoL).ti,ab,kw.	13
32	("Glasgow Epilepsy Outcome Scale" or "GEOS-YP").ti,ab,kw.	9
33	"Impact of Epilepsy Schedule".ti,ab,kw.	1
34	(QOLIE or QOLIE-31 or QOLIE-10).ti,ab,kw.	1092
35	"Pediatric Quality of Life Inventory or PedsQL".ti,ab,kw.	0
36	21 or 22 or 23 or 24 or 25 or 26 or 27 or 28 or 29 or 30 or 31 or 32 or 33 or 34 or 35	16162
37	(15D or 15-D or 15 dimension).ti,ab,kw.	7059
38	(eq-5d or eq5d or eq-5 or eq5 or EQ-5D-Y or euro qual or euroqual or euro qual5d or euroqual5d or euro qol or euroqol or euro qol5d or euroqol5d or euro quol or euroquol or euro quol5d or euroquol5d or eur qol or eurqol or eur qol5d or eur qol5d or eur?qul or eur?qul5d or euro$ quality of life or european qol or EQ-5D-3L).ti,ab,ot,hw,kw.	26595
39	(sf6 or sf 6 or SF-6D or short form 6 or short-form 6 or short-form six or shortform 6 or sf six or sfsix or shortform six or short form six).ti,ab,ot,hw,kw.	3949
40	(sf10 or sf 10 or short form 10 or short-form 10 or short-form ten or shortform 10 or sf ten or sften or shortform ten or short form ten).ti,ab,ot,hw,kw.	224
41	(sf12 or sf 12 or short form 12 or short-form 12 or short-form twelve or shortform 12 or sf twelve of sftwelve or shortform twelve or short form twelve).ti,ab,ot,hw,kw.	12504
42	(sf16 or sf 16 or short form 16 or short-form 16 or short-form sixteen or shortform 16 or sf sixteen or sfsixteen or shortform sixteen or short form sixteen).ti,ab,ot,hw,kw.	65
43	(sf20 or sf 20 or short form 20 or short-form 20 or short-form twenty or shortform 20 or sf twenty of sftwenty or shortform twenty of short form twenty).ti,ab,ot,hw,kw.	517
44	(sf36 or sf 36 or short form 36 or short-form 36 or short-form thirty six or shortform 36 or sf thirtysix or sf thirty six or shortform thirstysix or shortform thirty six or short form thirty six or short form thirtysix or short form thirty six).ti,ab,ot,hw,kw.	52721
45	(health utilities index$ or (hui or hui1 or hui2 or hui3 or hui4 or hui-4 or hui-1 or hui-2 or hui-3)).ti,ab,ot,hw,kw.	3744

46	("time trade off" or "time tradeoff" or "time trade-off" or TTO).ti,ab,ot,hw,kw.	3028
47	(standard gamble$ or SG).ti,ab,ot,hw,kw.	17941
48	("discrete choice" or DCE).ti,ab,ot,hw,kw.	12285
49	(AQoL or "Assessment of Quality of Life").ti,ab,ot,hw,kw.	3383
50	Quality-Adjusted Life Years/	29703
51	(HRQoL or HRQL or HQL or QoL or (quality adj3 life) or HYE or HYES or (health$ adj3 year$)).ti,ab,ot,hw,kw.	719594
52	"quality of life"/	520565
53	socioeconomics/	142119
54	uncertainty/	37234
55	(uncertain$ or wellbeing or "well being" or rosser or "willingness to pay").tw.	377239
56	(utilit$ or disutility$).ti,ab,kw.	319447
57	(illness state$1 or health state$ or health status or Quality adjusted life year$ or QALY or QALD or qale or qtime or life year$ or ICER or "incremental cost").ti,ab,ot,hw,kw.	223480
58	(burden and (disease or illness or caregiver or home)).tw.	175208
59	(lost adj2 (productivity or work or employment or earnings)).ti,ab,kw.	4554
60	(((disability or diseas$) adj3 adjust$) or daly$).ti,ab,kw.	14646
61	(preference* adj3 (valu* or measur* or health or life or estimat* or elicit* or disease or score* or instrument or instruments)).ti,ab,kw.	15912
62	(self report$ or (patient adj report$ adj outcome$)).ti,ab,kw.	268351
63	(mortality or death).ti,ab,kw.	2073283
64	37 or 38 or 39 or 40 or 41 or 42 or 43 or 44 or 45 or 46 or 47 or 48 or 49 or 50 or 51 or 52 or 53 or 54 or 55 or 56 or 57 or 58 or 59 or 60 or 61 or 62 or 63	3866328
65	36 or 64	3878406
66	7 or 20 or 65	7318863
67	3 and 66	655
68	(conference abstract or conference review or Conference paper).pt.	4939746
69	67 and 68	217
70	(2018* or 2019* or 2020* or 2021*).yr.	6288177
71	69 and 70	40
