# Supplementary material for: The burden of illness in Lennox–Gastaut syndrome: a systematic literature review
Source: Orphanet J Rare Dis. 2023 Mar 1;18:42. doi: 10.1186/s13023-023-02626-4 (PMC9979426; doi:10.1186/s13023-023-02626-4)
Supplement: Supplementary file 2 — Additional file 2. Supplementary Table S1: Healthcare resource utilization and costs in patients with LGS; Table S2: Quality assessment checklist for prevalence studies; Table S3: Quality assessment checklist of cost-of-illness studies; Table S4: Quality assessment checklist for qualitative HRQoL studies; Table S5: HRQoL: Quality assessment checklist for quantitative HRQoL studies. [file 13023_2023_2626_MOESM2_ESM.pdf]

Supplementary Table S1: Healthcare resource utilization and costs in patients with LGS

Study, country & type of study	Patient population	Healthcare resource	Costs
Piña-Garza 2017 [35]<br>USA<br>(Full)<br>Retrospective insurance claims analysis	Probable LGS patients (N=14,712) vs non-LGS patients (N=353,281)	*Mean (SD) in LGS vs non-LGS (PPPY)*<br>Outpatient visits: 11.8 (24.1) vs 9.0 (20.7)<br>Inpatient visits: 0.6 (1.5) vs 0.6 (1.7)<br>Emergency department visits: 1.4 (4.5) vs 1.3 (4.1)<br>EEG procedures: 0.3 (0.7) vs 0.1 (0.4)<br>Neurological procedures: 1.0 (1.9) vs 0.3 (1.0)<br>Use of wheelchair or walker: 0.1 (0.4) vs 0.0 (0.4)<br>ASMs:<br>Number of ASMs received: 5.8 (2.3) vs 1.8 (1.3)<br>≥1 claim for an ASM: 62.6%–82.3%	*Mean, LGS vs non-LGS (PPPY)*<br>Total healthcare costs: $28,461–$40,193 vs $7170–$25,901<br>*LGS: Costs across 10-year age cohorts PPPY*<br>Medical costs: $25,303–$37,342<br>Home-based care: 1-18 years: $12,396 to $18,360; 60-year-old cohort: $6978<br>Long-term care: 1-18 years: $1648 to $5933; 60-year-old cohort: $16,215<br>Pharmacy costs: $1592–$5630
Strzelczyk 2021 [36]<br>Germany<br>(Full)<br>Retrospective insurance claims analysis	Narrow LGS (N=208; 1379 patient-years)	Annual hospital admissions (PPPY): mean (SD; median [range]): 1.6 (2.0; 1 [0–13])<br><br>LOS: mean (SD; median [range]) (PPPY): 22.7 (46.0; 3 [0–804]) days	*Mean (PPPY)*<br>Total cost of healthcare: €22,787<br>Inpatient care: €7422 (proportion of total heathcare costs: 33%)<br>Outpatient care: €1390 (6%)<br>Medication: €2243 (10%)<br>ASMs alone: €309<br>Services and devices: €11,731 (51%)<br>Intensive home nursing care (€1971) and home nursing care (€985): €2956 (13%)
Chin 2021 [29]<br>UK<br>(Full)<br>Retrospective analysis of electronic medical records from healthcare databases	Confirmed LGS<br>Primary care <12 years (N=39) vs ≥ 12 years (N=89)<br>Secondary care <12 years (N=20) vs ≥ 12 years (N=55)<br><br>Probable LGS<br>Primary care <12 years (N=71) vs ≥ 12 years (N=115)<br>Secondary care <12 years (N=23) vs ≥ 12 years (N=47)	*Mean (SD) < 12 years vs ≥ 12 years (PPPY)*<br>*Confirmed LGS*<br>Primary care consultations: 6.46 (4.82) vs 6.79 (7.19)<br>Hospital outpatient visits: 7.45 (9.51) vs 5.36 (7.61)<br>Hospital inpatient admissions:<br>-All cause: 1.65 (1.63) vs 1.09 (1.86)<br>- Epilepsy-related: 1.50 (1.47) vs 0.96 (1.78)<br>ED visits: 0.85 (1.18) vs 1.15 (2.17)<br><br>LOS, days<br>-All causes: 2.41 (5.87) vs 3.42 (8.53) days<br>- Epilepsy-related: 2.48 (6.07) vs 3.24 (6.80)<br><br>Mean (SD) number of ASMs over follow-up period: 6.7 (3.4)<br>Mean (SD [range]) number of ASMs per year: 1.06 (0.27 [0.25–1.5]) during 2010–13 and 1.12 (0.39 [0.25–2.0]) during 2014–17<br><br>*Probable LGS*<br>Primary care consultations: 7.75 (5.37) vs 7.99 (7.08)<br>Hospital outpatient visits: 10.04 (10.49) vs 7.13 (7.48)	NR

Study, country & type of study	Patient population	Healthcare resource	Costs
		Hospital inpatient admissions:<br>-All cause: 3.61 (4.85) vs 1.26 (2.06)<br>- Epilepsy-related: 3.04 (4.43) vs 0.89 (1.37)<br>A&E visits: 0.96 (1.69) vs 1.04 (2.54)<br><br>LOS, days<br>-All causes: 3.53 (11.06) vs 4.74 (11.94) days<br>- Epilepsy-related: 3.69 (11.98) vs 5.70 (13.90)<br><br>Mean (SD [range]) number of ASMs per year: 1.06 (0.27 [0.25–1.5]) during 2010–13 and 1.22 (0.48 [0.25–3.25]) during 2014–17	
Reaven 2018 [43]<br>USA<br>(Full)<br>Retrospective insurance claims analysis	*Commercial:* Probable LGS patients (N=2270) vs control (N=2270)<br>*Medicaid:* Probable LGS patients (N=3749) vs control (N=3749)<br>Control= Patients lacking diagnoses of epilepsy or seizures and outpatient claims for any selected ASMs matched for age, sex, one of four US geographic regions, and insurance type.	*Mean (SD) in LGS vs control (PPPY)*<br><br>*Commercial: Probable LGS patients vs control*<br>(p<0.0001 for all comparisons)<br>Inpatient admissions: 0.7 (1.1) vs 0 (0.1)<br>ED visits w/o Admission: 1.1 (1.6) vs 0.2 (0.5)<br>Hospital OP visits: 8.2 (22.2) vs 0.5 (1.3)<br>Physician visits: 13.5 (19.6) vs 3.8 (5.7)<br>Other OP: 9.6 (19.6) vs 1.5 (4.4)<br>Home health: 16.2 (46.8) vs 0.1 (1.5)<br>Equipment/ supply: 4.0 (9.3) vs 0.1 (0.4)<br>ASMs: 23.1 (14.5) vs 0.0 (0.3)<br>Rescue ASMs: 1.5 (4.4) vs 0.0 (0.0)<br>Other drugs: 22.3 (26.1) vs 3.8 (6.1)<br>Total services: 53.4 (71.2) vs 6.2 (9.0)<br>Total drugs: 47.0 (34.9) vs 3.8 (6.2)<br><br><br>*Medicaid: Probable LGS patients vs control*<br>(p<0.0001 for all comparisons)<br>Inpatient admissions: 0.6 (0.9) vs 0.1 (0.3)<br>ED visits w/o Admission: 1.4 (2.1) vs 0.6 (1.2)<br>Hospital OP visits: 6.1 (14.8) vs 0.9 (3.3)<br>Physician visits: 8.0 (10.0) vs 3.4 (6.6)<br>Other OP: 48.9 (81.5) vs 7.0 (23.6)<br>Home health: 81.2 (126.0) vs 2.8 (22.4)<br>Equipment/ supply: 2.5 (6.0) vs 0.2 (1.0)<br>ASMs: 28.4 (16.1) vs 0.1 (1.0)<br>Rescue ASMs: 1.9 (4.2) vs 0.0 (0.1)<br>Other drugs: 35.6 (40.2) vs 8.2 (15.8)<br>Total services: 148.6 (156.1) vs 14.9 (37.0)<br>Total drugs: 66.0 (48.0) vs 8.3 (16.2)	*Mean (SD) in LGS vs control (PPPY)*<br><br>*Commercial: Probable LGS patients vs control*<br>(p<0.0001 for all comparisons)<br>Inpatient admissions: $22,907 (50,800) vs $460 (6482)<br>ED visits w/o Admission: $2149 (4602) vs $275 (813)<br>Hospital OP visits: $10,330 (23,548) vs $541 (2123)<br>Physician visits: $2966 (10,552) vs $585 (870)<br>Other OP: $2283 (7,160) vs $181 (652)<br>Home health: $8569 (38,148) vs $19 (340)<br>Equipment/ supply: $1843 (5,044) vs $20 (192)<br>ASMs: $8531 (13,667) vs $1 (14)<br>Rescue drugs: $755 (3071) vs $0 (0)<br>Other drugs: $4693 (18,306) vs $361 (1433)<br>Total services: $51,047 (83,203) vs $2081 (7639)<br>Total drugs: $13,979 (23,999) vs $362 (1433)<br>Total cost: $65,026 (91,006) vs $2442 (7950)<br><br>*Medicaid: Probable LGS patients vs control*<br>(p<0.0001 for all comparisons)<br>Inpatient admissions: $12,815 (40,250) vs $870 (8288)<br>ED visits w/o Admission: $838 (1852) vs $230 (728)<br>Hospital OP visits: $3526 (16,811) vs $304 (1,242)<br>Physician visits: $836 (2087) vs $277 (802)<br>Other OP: $9362 (24,471) vs $825 (3,839)<br>Home health: $23,725 (42,365) vs $610 (4748)<br>Equipment/ supply: $1334 (2683) vs $31 (385)<br>ASMs: $6566 (11,869) vs $3 (77)<br>Rescue drugs: $809 (2520) vs $0 (4)<br>Other drugs: $4118 (15,818) vs $699 (4482)<br>Total services: $52,437 (69,462) vs $3147 (11,487)<br>Total drugs: $11,493 (21,684) vs $702 (4485)

Study, country & type of study	Patient population	Healthcare resource	Costs
			Total costs: $63,930 (76,929) vs $3849 (13,703)
Reaven 2019 [42]<br>(LGS seizure events vs no events<br>USA<br>(Full)<br>Retrospective insurance claims analysis	Probable LGS:<br>*Commercial:* Patients with seizure events (N=1258) vs no seizure events (N=1011)<br>*Medicaid:* Patients with seizure events (N=2192) vs no event (N=1538)	*Mean (SD) in LGS patients with events vs no events (PPPY)*<br>*Commercial:*<br>Inpatient admissions: 1.1 (1.3) vs 0.3 (0.5)<br>ED visits: 1.7 (1.9) vs 0.3 (0.6)<br>Hospital OP visits: 10.1 (25.2) vs 5.9 (17.4)<br>Physician visits: 15.4 (21.9) vs 11.1 (15.8)<br>Other OP: 10.9 (20.8) vs 8.0 (17.9)<br>Home health: 18.1 (50.2) vs 13.8 (42.1)<br>Equipment/ supply: 4.5 (10.1) vs 3.4 (8.1)<br>ASMs: 24.4 (15.1) vs 21.6 (13.7)<br>Rescue ASMs: 2.0 (5.1) vs 0.9 (3.1)<br>Other drugs: 24.9 (28.5) vs 19.2 (22.2)<br>*Medicaid:*<br>Inpatient admissions: 0.8 (1.4) vs 0.2 (0.4)<br>ED visits: 2.1 (2.4) vs 0.4 (0.0)<br>Hospital OP visits: 7.4 (17.0) vs 4.4 (11.0)<br>Physician visits: 9.0 (10.8) vs 6.6 (8.0)<br>Other OP: 48.6 (81.8) vs 49.7 (81.5)<br>Home health: 76.5 (122.7) vs 88.7 (130.6)<br>Equipment/ supply: 2.7 (6.4) vs 2.3 (5.5)<br>ASMs: 29.1 (16.4) vs 27.7 (15.0)<br>Rescue ASMs: 2.3 (4.5) vs 1.3 (3.6)<br>Other drugs: 37.2 (41.9) vs 33.6 (37.8)	*Mean (SD) in LGS patients with events vs no events (PPPY)*<br>*Commercial:*<br>Inpatient admissions: $34,929 (67,542) vs $9555 (33,644)<br>ED visits: $3469 (5748) vs $505 (1241)<br>Hospital OP visits: $12,841 (27,857) vs $7491 (18,874)<br>Physician visits: $3483 (12,874) vs $2337 (6550)<br>Other OP: $2917 (8837) vs $1546 (4253)<br>Home health: $9722 (39,765) vs $7215 (36,046)<br>Equipment/ supply: $1994 (5502) vs $1663 (4403)<br>ASMs: $9144 (14,464) vs $7756 (12,559)<br>Rescue drugs: $974 (3395) vs $482 (2585)<br>Other drugs: $5467 (18,431) vs $3742 (18,096)<br>Total services: $69,354 (101,479) vs $30,311 (63,955)<br>Total drugs: $15,585 (25,239) vs $11,980 (22,184)<br>Total costs: $84,939 (109,786) vs $42,292 (69,147)<br>*Medicaid:*<br>Inpatient admissions: $20,681 (61,771) vs $5318 (43,352)<br>ED visits: $1272 (1898) vs $214 (1493)<br>Hospital OP visits: $4208 (20,956) vs $2551 (4756)<br>Physician visits: $962 (2440) vs $724 (1890)<br>Other OP: $9144 (25,210) vs $8952 (21,809)<br>Home health: $21,492 (39,840) vs $26,191 (44,588)<br>Equipment/ supply: $1346 (2661) vs $1378 (2763)<br>ASMs: $6914 (12,982) vs $5882 (9672)<br>Rescue drugs: $980 (2640) vs $574 (2334)<br>Other drugs: $4512 (18,923) vs $3655 (9407)<br>Total services: $59,106 (86,008) vs $45,329 (67,406)<br>Total drugs: $12,406 (25,184) vs $10,110 (14,824)<br>Total costs: $71,512 (94,213) vs $55,439 (71,687)
Reaven 2019 [42]<br>(LGS vs other DEEs)<br>USA<br>(Full)<br>Retrospective insurance claims analysis	*Commercial:* Probable LGS (N=2269) vs DS (N=321) vs TSC (N=1622)<br><br>*Medicaid:* Probable LGS (N=3730) vs DS (N=668) vs TSC (N=1144)	*Mean (SD) in probable LGS vs DS vs TSC (PPPY)*<br><br>*Commercial:*<br>Inpatient admissions: 0.7 (1.1) vs 0.5 (0.8) vs 0.2 (0.4)<br>ED visits: 1.1 (1.6) vs 1.0 (1.5) vs 0.4 (0.9)<br>Hospital OP visits: 8.2 (22.2) vs 7.0 (20.3) vs 3.0 (5.9)<br>Physician visits: 13.5 (19.6) vs 10.7 (11.5) vs 9.1 (12.7)<br>Other OP: 9.6 (19.6) vs 9.1 (20.1) vs 5.8 (15.1)<br>Home health: 16.2 (46.8) vs 5.7 (20.0) vs 1.2 (10.6)<br>Equipment/ supply: 4.0 (9.3) vs 1.0 (3.0) vs 0.4 (1.8)<br>ASMs: 23.1 (14.5) vs 18.9 (11.5) vs 5.4 (9.9)<br>Rescue ASMs: 1.5 (4.4) vs 1.0 (3.2) vs 0.1 (0.6)<br>Other drugs: 22.3 (26.1) vs 15.0 (16.3) vs 11.4 (17.1)<br>*Medicaid:*<br>Inpatient admissions: 0.6 (1.0) vs 0.4 (0.7) vs 0.3 (0.7)<br>ED visits: 1.4 (2.1) vs 1.4 (2.1) vs 1.0 (1.9)	*Mean (SD) in probable LGS vs DS vs TSC (PPPY)*<br>*Commercial:*<br>Inpatient admissions: $23,623 (56,494) vs $10,847 (28,133) vs $5226 (19,684)<br>ED visits: $2148 (4601) vs $1811 (3290) vs $902 (2892)<br>Hospital OP visits: $10,458 (24,409) vs $5769 (11,643) vs $4951 (9795)<br>Physician visits: $2972 (10,549) vs $2154 (3255) vs $1885 (4828)<br>Other OP: $2306 (7197) vs $1817 (4479) vs $1210 (9963)<br>Home health: $8605 (38,165) vs $1540 (8445) vs $475 (7116)<br>Equipment/ supply: $1846 (5044) vs $438 (1644) vs $141 (1230)<br>ASMs: $8526 (13,662) vs $4130 (5375) vs $3087 (12,019)<br>Rescue drugs: $755 (3070) vs $516 (2013) vs $64 (390)

Study, country & type of study	Patient population	Healthcare resource	Costs
		Hospital OP visits: 6.1 (14.9) vs 3.8 (8.3) vs 3.8 (6.8)<br>Physician visits: 8.0 (10.0) vs 6.4 (9.4) vs 7.5 (16.3)<br>Other OP: 49.1 (81.0) vs 43.1 (84.2) vs 44.9 (90.2)<br>Home health: 81.5 (126.2) vs 44.4 (97.8) vs 34.0 (84.2)<br>Equipment/ supply: 2.5 (6.0) vs 0.7 (2.7) vs 0.8 (2.7)<br>ASMs: 28.5 (16.1) vs 21.3 (13.1) vs 11.6 (14.9)<br>Rescue ASMs: 1.9 (4.2) vs 1.0 (2.4) vs 0.4 (1.2)<br>Other drugs: 35.7 (40.3) vs 27.7 (35.9) vs 26.6 (37.6)	Other drugs: $4698 (18,299) vs $2411 (8020) vs $2965 (14,713)<br>Total services: $51,958 (88,914) vs $24,376 (38,308) vs $14,790 (32,935)<br>Total drugs: $13,979 (23,987) vs $7057 (9962) vs $6117 (20,148)<br>Total costs: $65,937 (96,223) vs $31,433 (41,835) vs $20,907 (40,657)<br>*Medicaid:*<br>Inpatient admissions: $14,346 (55,442) vs $5694 (23,135) vs $4062 (17,487)<br>ED visits: $836 (1819) vs $810 (1647) vs $520 (1482)<br>Hospital OP visits: $3525 (16,371) vs $1728 (1647) vs $2086 (5299)<br>Physician visits: $864 (2233) vs $574 (961) vs $767 (3448)<br>Other OP: $9065 (23,864) vs $7566 (20,646) vs $8178 (23,402)<br>Home health: $23,430 (41,921) vs $10,390 (26,767) vs $8061 (23,815)<br>Equipment/ supply: $1359 (2703)) vs $276 (1076) vs $203 (931)<br>ASMs: $6488 (11,740) vs $2488 (4440) vs $3150 (10,815)<br>Rescue drugs: $813 (2526) vs $360 (1038) vs $142 (626)<br>Other drugs: $4159 (15,717) vs $1998 (4726) vs $4115 (18,875)<br>Total services: $53,425 (79,152) vs $27,039 (42,876) vs $23,878 (38,111)<br>Total drugs: $11,460 (21,552) vs $4845 (6904) vs $7407 (23,252)<br>Total costs: $64,885 (86,000) vs $31,884 (45,174) vs $31,284 (46,777)
Stockl 2019 [45]<br>USA<br>(Abstract)<br>Retrospective insurance claims analysis	Probable LGS:<br>*Commercial* (N=2520), *Medicaid* (N=4613)	Hospitalizations by healthplan:<br>Epilepsy-related index-hospitalizations: 46–58%<br>Pneumonia-related index hospitalizations: 6–7%<br>Injury-related index-hospitalizations: 2%*<br>ICU use: 31%*<br>Mean (SD) LOS ICU vs non-ICU use: 8.0 (16.8) vs 4.0 (7.9) days*<br>* Data are for all patients (LGS+DS+TSC)	NR
Stockl 2019 [44]<br>USA<br>(Abstract)<br>Retrospective insurance claims analysis	Probable LGS (N=1296) vs probable DS (N=183) vs other DEEs (N=6717)	*Commercial:* Probable LGS vs probable DS vs other DEEs<br>Number of distinct ASMs during the 12-month pre-index period: 3.4 vs 2.6 vs 2.1	*Commercial:* Probable LGS vs probable DS vs other DEEs<br><br>Mean costs 12-month post-index following each patient’s earliest diagnosis or ASM claim (index date)

Study, country & type of study	Patient population	Healthcare resource	Costs
			All-cause total healthcare costs: $80,545 vs $77,914 vs $43,794<br>All-cause medical costs: $56,527 vs $63,850 vs $32,403<br>-Proportion epilepsy related costs: 71.2% vs 80.5% vs 62.5%<br>Pharmacy costs: $24,018 vs $14,064 vs $11,391<br>-Proportion ASM costs: 72.6% vs 70.8% vs 65.8%
Hollenack 2019 [41]<br>USA<br>(Abstract)<br>Retrospective insurance claims analysis	Probable LGS *(*N=5186) vs probable DS (N=504) vs other DEEs (N=9453)	*Medicaid:* Probable LGS vs probable DS vs other DEEs<br><br>Number of distinct ASMs during the 12-month pre-index period: 2.4 vs 2.3 vs 2.2	*Medicaid:* Probable LGS vs probable DS vs other DEEs<br><br>Mean costs 12-month post-index following each patient's earliest diagnosis or ASM claim (index date)<br><br>All-cause total healthcare costs: $49,304 vs $31,342 vs $33,183<br>All-cause medical costs: $36,356 vs $22,790 vs $23,650<br>-Proportion epilepsy related costs: 31.5% vs 27.3% vs 48.0%<br>Pharmacy costs: $12,948 vs $8,551 vs $9,533<br>-Proportion ASM costs: 67.3%, 62.8%, 52.3%
François 2017 [40]<br>USA<br>(Full)<br>Retrospective insurance claims analysis	LGS (prior to clobazam initiation)<br>*Commercial and medicare (*N=1384), *Medicaid (*N=1365)	*Mean (SD)) in commercial & medicaid (PPPY)*<br>*Seizure related*<br>Hospitalization: 0.4 (0.8) & 0.3 (0.8)<br>-LOS, days: 1.4 (4.7) & 1.4 (6.0)<br>ED visits: 0.5 (1.0) & 0.9 (1.8)<br>Physician office visits: 2.2 (2.3) & 2.1 (2.2)<br>Laboratory visits: 0.3 (0.9) & 0.3 (0.9)<br>Radiology visits: 0.1 (0.3) & 0.1 (0.4)<br>Other outpatient: 1.8 (11.5) & 4.7 (27.6)<br><br>*All cause*<br>Hospitalization: 1.2 (1.8) & 1.1 (2.0)<br>-LOS, days: 6.5 (18.2) & 6.7 (19.8)<br>ED visits: 2.1 (3.2) & 3.4 (5.2)<br>Physician office visits: 10.2 (7.6) & 8.9 (7.2)<br>Laboratory visits: 1.9 (3.0) & 1.8 (3.5)<br>Radiology visits: 1.4 (2.9) & 1.0 (1.7)<br>Other outpatient: 18.1 (31.3) & 63.9 (87.7)	*Mean (SD)) in commercial & medicaid (PPPY)*<br>*Seizure related*<br>Total: $12,709 (36,420) & $7687 (25,092)<br>Medical: $10,563 (35,194) & $5951 (24,374)<br>-Hospitalization: $7366 (30,951) & $4108 (22,651)<br>-ED visits: $793 (2314) & $353 (979)<br>-Physician office visits: $562 (1438) & $246 (699)<br>-Laboratory visits: $82 (621) & $16 (98)<br>-Radiology visits: $126 (632) & $43 (212)<br>-Other outpatient: $1634 (11,555) & $1186 (8086)<br>Prescription: $2146 (5596) & $1736 (4262)<br><br>*All cause*<br>Medical: $43,866 (86,376) & $34,292 (68,737)<br>-Hospitalization: $24,727 (70,535) & $14,498 (58,921)<br>-ED visits: $4671 (9646) & $2036 (4009)<br>-Physician office visits: $2841 (5036) & $1579 (2738)<br>-Laboratory visits: $369 (1163) & $101 (253)<br>-Radiology visits: $930 (3911) & $224 (586)<br>-Other outpatient: $10,329 (27,363) & $15,854 (28,874)<br>Prescription: $5766 (18,940) & $4078 (7315)

ASM, anti-seizure medication; DEE, developmental and epileptic encephalopathy; DS, Dravet syndrome; ED, emergency department; ICU, intensive care unit; LGS, Lennox-Gastaut syndrome; LOS, length of stay; NA, non-applicable; OP, outpatient; PPPY, per person per year; SD, standard deviation; TSC, tuberous sclerosis complex

Table S2: Quality assessment checklist for prevalence studies

	**Strzelczyk 2021 [36] (Full)**	**Chin 2021 [29] (Full)**	**Hollenack 2019 [30] (Abstract)**	**MADDS study Trevathan 1997 [33] (Full)**	**Sidenvall 1996 [32] (Full)**	**Rantala 1999 [31] (Full)**	**Heiskala 1997 [34] (Full)**	**Beilmann 1999 [28] (Full)**
Was the study's target population a close representation of the national population in relation to relevant variables, e.g. age, sex, occupation?	0	0	0	0	1	1	1	0
Was the sampling frame a true or close representation of the target population?	0	0	0	1	1	1	1	1
Was some form of random selection used to select the sample, OR, was a census undertaken?	0	0	0	1	1	1	1	1
Was the likelihood of non-response bias minimal?	1	1	1	1	1	1	1	1
Were data collected directly from the subjects (as opposed to a proxy)?	0	0	0	1	1	1	1	1
Was an acceptable case definition used in the study?	1	1	1	1	1	1	1	1
Was the study instrument that measured the parameter of interest (e.g. prevalence of low back pain) shown to have reliability and validity (if necessary)?	2	2	2	1	1	1	1	1
Was the same mode of data collection used for all subjects?	0	0	0	1	1	1	1	1
Were the numerator(s) and denominator(s) for the parameter of interest appropriate?	1	1	1	1	1	1	1	1
Overall risk of bias<br>Low risk=0-6<br>Moderate =7-12<br>High =13-18	5=low	5=low	5=low	8=moderate	9=moderate	9=moderate	9=moderate	8=moderate

From Hoy et al [24]; 0=low risk; 1= moderate/unclear; 2=high risk

Table S3: Quality assessment checklist of cost-of-illness studies

	Piña-Garza 2017 [35]	Strzelczyk 2021 [36] (Full)	Chin 2021 [29] (Full)	Reaven 2018 [43] (Full)	Reaven 2019 [42] (Full)	Stockl 2019 [45] (Abstract)	Stockl 2019 [44] (Abstract)	Hollenack 2019 [41] (Abstract)	François 2017 [40] (Full)
Was a clear definition of the illness given?	P	P	P	P	P	P	P	P	P
Were epidemiological sources carefully described?	1	1	1	1	1	1	1	1	1
Were direct/indirect costs/ resource sufficiently disaggregated?	P	P	P	P	P	P	P	P	P
Were activity data sources carefully described?	1	1	1	1	1	P	P	P	1
Were activity data appropriately assessed?	1	1	1	1	1	P	P	P	1
Were the sources of all cost values analytically described?	P	P	P	P	P	0	0	0	P
Were unit costs appropriately valued?	1	1	1	1	1	1	1	1	1
Were the methods adopted carefully explained?	1	1	1	1	1	0	0	0	1
Were the major assumptions tested in a sensitivity analysis?	0	0	0	0	0	0	0	0	0
Was the presentation of study results consistent with the methodology of the study?	1	1	1	1	1	1	1	1	1
Total score by study									
YES(1)=low risk	**6**	**6**	**6**	**6**	**6**	3	3	3	**6**
NO(0)=high risk	1	1	1	1	1	3	3	3	1
PARTIALLY(p)=moderate risk	3	3	3	3	3	**4**	**4**	**4**	3

From Molinier et al.[26] Mostly1=low risk; P= moderate/unclear; 0=high risk

Table S4: Quality assessment checklist for qualitative HRQoL studies

	Appraisal/score	Gallop 2010 [55] (Full)	Murray 1993 [49] (Full)	Gibson 2014 [48] (Full)
Addresses a research question closely related to our review aims	Yes/No<br>Only "yes" can be grade I or II	Yes	Yes	Yes
Qualitative methods are appropriate for the research question	Yes=3<br>No=0	3	3	3
Details of caregiver (relationship to patient, age, gender) and patient features (age, seizure frequency/ disease severity) reported	Both caregiver and patient = 3<br>Only one = 1<br>Neither = 0	3	0	0
Methods described in sufficient detail (e.g., how participants were recruited, what did the interview guide ask, etc.)	Yes = 3<br>Partial = 1<br>No = 0	3	1	1
Analysis described in sufficient detail (analysis approach e.g., grounded theory/thematic analysis, analysis procedures, saturation assessed) [yes/no score: 0/2]	Yes = 3<br>Partial = 1<br>No = 0	3	1	1
Caregiver quotes included	Yes = 1<br>No = 0	1	1	1
Reports ethical review/approval	Yes = 1<br>No = 0	1	0	0
Evidence of obvious bias in methodology (e.g., recruitment bias, focused on one treatment)	No obvious bias = 3<br>Some evidence of bias = 1<br>Several sources of bias = 0	1	1	1
Total score range	13–17 Grade I (if 'yes' to first question)<br><br>8–12 Grade II<br><br>0–7 Grade III	Grade I	Grade III	Grade III

From Gallop et al [27] Grade I= highest methodological and reporting quality; Grade 2= moderate-high methodological and reporting quality; Grade 3= limitations in their methodological and reporting quality

Table S5: HRQoL: Quality assessment checklist for quantitative HRQoL studies

	Appraisal/score	Auvin 2021 [50] (Full)	Radu 2019 [51] (Abstract)	Gallop 2010 [55] (Full)
Addresses a research question closely related to our review aims	Yes/No<br>Only "yes" can be grade I or II	Yes	Yes	Yes
Validated questionnaires (e.g., EQ-5D, ZBI, CarerQol, SF-36, GAD, BDI)	Validated questionnaires = 3<br>Well-described bespoke survey = 1<br>Poorly described survey = 0	1	1	3
Good sample size	50+=3	3	1	1

	Appraisal/score	**Auvin 2021 [50] (Full)**	**Radu 2019 [51] (Abstract)**	**Gallop 2010 [55] (Full)**
	25–50=1<br>Less than 25=0			
Details of caregiver (relationship to patient, age, gender) and patient features (age, seizure frequency/ disease severity) reported	Both caregiver and patient = 3<br>Only one = 1<br>Neither = 0	1	0	3
Appropriate statistical reporting	Yes = 1<br>No = 0	1	1	1
Reports ethical review/approval	Yes = 1<br>No = 0	1	0	1
Evidence of obvious bias in methodology (e.g., recruitment bias, focused on one treatment)	No obvious bias = 3<br>Some evidence of bias = 1<br>Several sources of bias = 0	1	1	1
Total score range	11–14 Grade I (if 'yes' to first question)<br><br>7–10 Grade II<br><br>0–6 Grade III	Grade II	Grade III	Grade II

From Gallop et al [27] Grade I= highest methodological and reporting quality; Grade 2= moderate-high methodological and reporting quality; Grade 3= limitations in their methodological and reporting quality
