# Supplementary material for: The burden of illness in Lennox–Gastaut syndrome: a systematic literature review
Source: Orphanet J Rare Dis. 2023 Mar 1;18:42. doi: 10.1186/s13023-023-02626-4 (PMC9979426; doi:10.1186/s13023-023-02626-4)
Supplement: Supplementary file 3 — Additional file 3. Supplementary Figure S1: Costs, annual hospitalization rate and length of stay (LOS) in LGS patients in years where patients were prescribed with rescue medication vs years where patients were not prescribed rescue medication. [file 13023_2023_2626_MOESM3_ESM.pdf]

Supplementary Figure S1: Costs, annual hospitalization rate and length of stay (LOS) in LGS patients in years where patients were prescribed with rescue medication vs years where patients were not prescribed rescue medication

A

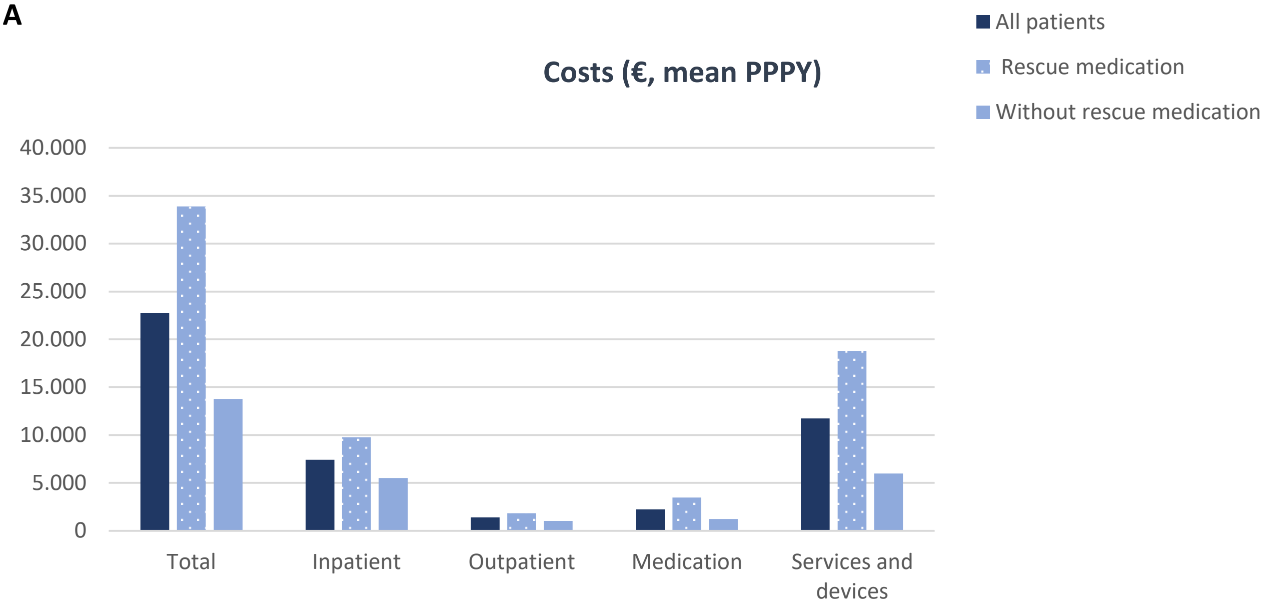

B

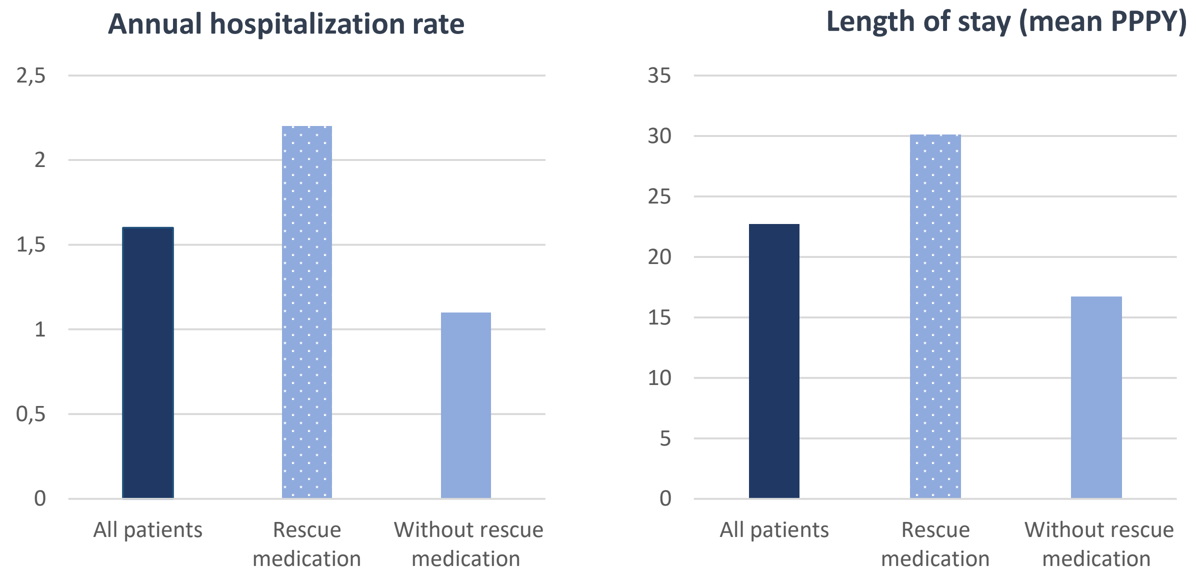

Adapted from: Strzelczyk 2021 [36]
